# The Glycosylphosphatidylinositol-Anchored *DFG* Family Is Essential for the Insertion of Galactomannan into the β-(1,3)-Glucan–Chitin Core of the Cell Wall of Aspergillus fumigatus

**DOI:** 10.1128/mSphere.00397-19

**Published:** 2019-07-31

**Authors:** Laetitia Muszkieta, Thierry Fontaine, Rémi Beau, Isabelle Mouyna, Marian Samuel Vogt, Jonathan Trow, Brendan P. Cormack, Lars-Oliver Essen, Gregory Jouvion, Jean-Paul Latgé

**Affiliations:** a Unité des Aspergillus, Institut Pasteur, Paris, France; b Faculty of Chemistry, Philipps-Universität Marburg, Marburg, Germany; c Department of Molecular Biology and Genetics, Johns Hopkins University School of Medicine, Baltimore, Maryland, USA; d Histopathologie humaine et modèles animaux, Institut Pasteur, Paris, France; Carnegie Mellon University

**Keywords:** *Aspergillus fumigatus*, cell wall, glycobiology

## Abstract

The fungal cell wall is a complex and dynamic entity essential for the development of fungi. It is composed mainly of polysaccharides that are synthetized by protein complexes. At the cell wall level, enzyme activities are involved in postsynthesis polysaccharide modifications such as cleavage, elongation, branching, and cross-linking. Glycosylphosphatidylinositol (GPI)-anchored proteins have been shown to participate in cell wall biosynthesis and specifically in polysaccharide remodeling. Among these proteins, the DFG family plays an essential role in controlling polar growth in yeast. In the filamentous fungus and opportunistic human pathogen Aspergillus fumigatus, the *DFG* gene family contains seven orthologous *DFG* genes among which only six are expressed under *in vitro* growth conditions. Deletions of single *DFG* genes revealed that *DFG3* plays the most important morphogenetic role in this gene family. A sextuple-deletion mutant resulting from the deletion of all *in vitro* expressed *DFG* genes did not contain galactomannan in the cell wall and has severe growth defects. This study has shown that DFG members are absolutely necessary for the insertion of galactomannan into the cell wall of A. fumigatus and that the proper cell wall localization of the galactomannan is essential for correct fungal morphogenesis in A. fumigatus.

**IMPORTANCE** The fungal cell wall is a complex and dynamic entity essential for the development of fungi. It is composed mainly of polysaccharides that are synthetized by protein complexes. Enzymes involved in postsynthesis polysaccharide modifications, such as cleavage, elongation, branching, and cross-linking, are essential for fungal life. Here, we investigated in Aspergillus fumigatus the role of the members of the Dfg family, one of the 4 GPI-anchored protein families common to yeast and molds involved in cell wall remodeling. Molecular and biochemical approaches showed that DFG members are required for filamentous growth, conidiation, and cell wall organization and are essential for the life of this fungal pathogen.

## Abstract (precis)

The fungal cell wall is a complex and dynamic entity essential for the development of fungi. It is composed mainly of polysaccharides that are synthetized by protein complexes. Enzymes involved in postsynthesis polysaccharide modifications, such as cleavage, elongation, branching, and cross-linking, are essential for fungal life. Here, we investigated in Aspergillus fumigatus the role of the members of the Dfg family, one of the 4 GPI-anchored protein families common to yeast and molds involved in cell wall remodeling. Molecular and biochemical approaches showed that DFG members are required for filamentous growth, conidiation, and cell wall organization and are essential for the life of this fungal pathogen.

## INTRODUCTION

The fungal cell wall is a complex and dynamic entity essential for the development of fungi. It has prominent and dual roles during the growth of fungal pathogens. It allows the pathogen to survive environmental challenges posed by nutrient stress, microbiota, or human cells, and it also is central to polarized growth, which helps the fungus to invade host tissues (1). The cell wall of Aspergillus fumigatus is mainly composed of polysaccharides organized in a three-dimensional (3D) network (2). Enzymes involved in the biosynthesis of linear glucan and chitin, the main cell wall polysaccharides encountered in the fungal kingdom, have been identified previously (3–5). However, the transglycosidases responsible for the branching and cross-linking of these linear polysaccharides in a 3D rigid skeleton are only beginning to be discovered (6). Early studies have shown the merits of concurrent analyses of putative transglycosylases in different fungal species such as Saccharomyces cerevisiae and Aspergillus fumigatus with different cell wall compositions but similar central polysaccharide 3D cores. Such analyses has led to the selection of four groups of glycosylphosphatidylinositol (GPI)-anchored proteins common to all fungi which could potentially have a central role in the transglycosylation of the cell wall structural polysaccharides. First, a family of β-(1,3)-glucanosyltransferase activity discovered in A. fumigatus and encoded by the *GEL* gene or *GAS* gene is responsible for the elongation of the β-(1,3)-glucans and is essential in the biosynthesis of the cell wall (53, 54, 55). This family contains two subgroups that depend on the presence of a carbohydrate binding domain, which is responsible for a dual form of enzyme activity, β-glucan elongation and branching (6). Second, the Sps2p/Ecm33p family of GPI-anchored proteins has been described previously in both S. cerevisiae and A. fumigatus (7, 8). Members of the Sps2p family play an essential role in the formation of the ascospore cell wall in S. cerevisiae, whereas in A. fumigatus, Ecm33p is important for conidial morphogenesis and virulence. However, its enzymatic function remains unknown. Third, the Crhp family in S. cerevisiae is composed of three genes which are involved in the linkage of chitin to β-(1,6)-glucan in the cell wall (9, 10). Although also present in A. fumigatus, it is obvious that the Crh proteins do not have the same function in S. cerevisiae and A. fumigatus since there is no β-(1,6)-glucan in the cell wall of A. fumigatus. Finally, the two genes in the *DFG* (for “defective in filamentous growth”) family encode two GPI-anchored proteins with redundant activities in both S. cerevisiae and Candida albicans (11, 12). Although single knockouts of *DFG5* and *DCW1* are viable, a double knockout was synthetically lethal in both S. cerevisiae and C. albicans (11, 12). Cells depleted in either Dfg5p or Dcw1p released GPI-anchored cell wall proteins (GPI-CWPs) into the medium and showed increased cell volume, suggesting an alteration of the cell wall organization. However, the exact biochemical function of the Dfg proteins in yeast as well as in filamentous fungi remains unknown.

To understand the function of Dfg proteins in A. fumigatus, single-deletion and multiple *dfg* mutants targeting deletions of the members of the entire *DFG* family were constructed and analyzed. *DFG* multiple deletion resulted in the total loss of the cell wall galactomannan (GM), which was associated with severe growth phenotypes.

## RESULTS

### The DFG family in A. fumigatus.

**FIG 1:**
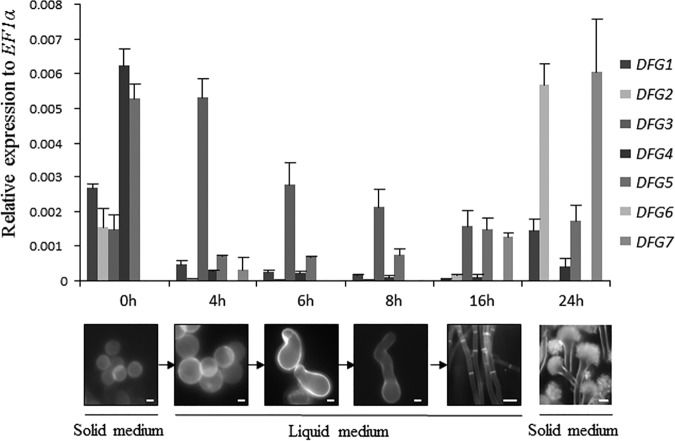
Expression profiles of seven *DFG* genes in different stages of A. fumigatus development assayed by qRT-PCR. RNA was extracted from freshly harvested resting (0 h), swollen (4 h), or germinated (6 to 8 h) conidia and mycelium grown in Sabouraud liquid medium and sporulating mycelium grown on Sabouraud solid medium. The relative expression levels of individual *DFG* genes were analyzed with the 2^ΔΔ^*^CT^* method with the EF1α gene used as an internal control for normalization. Microscopy analysis of developmental stages was followed by staining with calcofluor white. (Values represent means and standard deviations of results from three different experiments. Bars: time zero h [0h], 4h, 6h, and 8h, 1 μm; 16h, 10 μm; 24h, 20 μm.)

A BLAST query of the S. cerevisiae Dfg5p/Dcw1p protein sequences against the A. fumigatus genome database (https://fungi.ensembl.org/Aspergillus_fumigatusa1163/Info/Index) identified seven paralogs. The percentages of identity and similarity ranged between 15% and 42% and between 30% and 60%, respectively (see Table S1 in the supplemental material). *In silico* analysis revealed that all of the proteins encoded by these genes contained a secretory signal peptide at the N-terminal region (identified by the use of the SignalP website [http://www.cbs.dtu.dk/services/SignalP/]), a hydrophobic region at the C-terminal region with an ω-site characteristic of GPI-anchored proteins (except Dfg2p) (identified by the use of the BigPI website [http://mendel.imp.ac.at/gpi/fungi_server.html] and the PredGPI website [http://gpcr.biocomp.unibo.it/predgpi/pred.htm]), and the glycosyl hydrolase domain GH76 characteristic of Dfg5p/Dcw1p proteins from yeast (see Fig. S1 in the supplemental material). The expression levels of the *DFG* genes seen during different stages of development of A. fumigatus (swollen conidia, germinating conidia, and mycelium grown in Sabouraud liquid medium; sporulating mycelium and conidia cultivated on Sabouraud agar medium) were analyzed by quantitative real-time PCR (qRT-PCR) (Fig. 1). With the exception of *DFG*6, all of the *DFG* genes were expressed in the different fungal stages but their relative expression levels were highly dependent on the growth stage considered (Fig. 1). *DFG3* was the gene that was the most highly expressed in germinating conidia. Three *DFG* genes (*DFG1*, *DFG2*, and *DFG5*) were highly expressed in conidia and sporulating mycelium, while *DFG4* was highly expressed only in conidia and *DFG7* only in sporulating mycelium.

10.1128/mSphere.00397-19.2 FIG S1 Sequence alignment of the S. cerevisiae Dfg5p/Dcw1p and A. fumigatus Dfgp proteins. Protein sequences were aligned using ClustalW. The motifs conserved between different proteins are highlighted with light or dark characters on a light gray, dark gray, or black background. Download FIG S1, PPT file, 0.4 MB. Copyright © 2019 Muszkieta et al. 2019 Muszkieta et al. This content is distributed under the terms of the Creative Commons Attribution 4.0 International license.

10.1128/mSphere.00397-19.7 TABLE S1 Comparison of the *DFG* gene families in S. cerevisiae and A. fumigatus. Download Table S1, DOCX file, 0.01 MB. Copyright © 2019 Muszkieta et al. 2019 Muszkieta et al. This content is distributed under the terms of the Creative Commons Attribution 4.0 International license.

### Construction of Δ*dfg* mutants.

Based on the gene expression analysis and to further understand their biological role in fungal life, successive deletions of all members of the multigene *DFG* family in A. fumigatus were undertaken. These multiple deletions were carried out by employing the β-rec/six system (13). Single-deletion mutants (except for the nonexpressed *DFG6* gene) and multiple-deletion mutants (*Δdfg5*/*2*; *Δdfg5*/*2*/*1*; *Δdfg5*/*2*/*1*/*4*; *Δdfg5*/*2*/*1*/*3*; *Δdfg5*/*2*/*1*/*3*/*4*; *Δdfg5*/*2*/*1*/*3*/*4*/*7*) were constructed. Strategies for gene replacements and their validation are shown in Fig. S2, and the corresponding mutants are listed in Table S2.

10.1128/mSphere.00397-19.3 FIG S2 Targeted replacement strategies used for A. fumigatus *DFG* genes. Download FIG S2, PPT file, 1.2 MB. Copyright © 2019 Muszkieta et al. 2019 Muszkieta et al. This content is distributed under the terms of the Creative Commons Attribution 4.0 International license.

10.1128/mSphere.00397-19.8 TABLE S2 Aspergillus fumigatus **s**trains used in this study. Download Table S2, DOCX file, 0.02 MB. Copyright © 2019 Muszkieta et al. 2019 Muszkieta et al. This content is distributed under the terms of the Creative Commons Attribution 4.0 International license.

### Mycelial growth and hyphal morphology of Δ*dfg* mutants.

**FIG 2:**
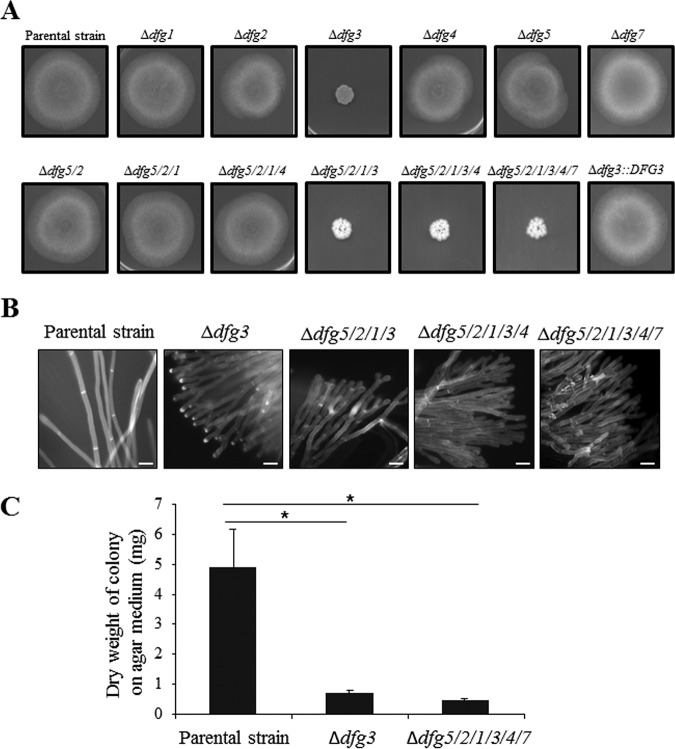
Growth of *DFG* deletion mutant strains on solid medium. (A) Radial growth of the parental and *DFG* deletion mutant strains on malt agar medium (48 h at 37°C). (B) Mycelial morphology of the parental strain and Δ*dfg* mutants grown on malt agar medium. Hyphae were stained with calcofluor white (bar, 10 μm). (C) Mycelial dry weight of the Δ*dfg mutants* obtained after 48 h of growth on malt agar medium at 37°C. (Values represent means and standard deviations of results from three different experiments; statistically significant differences [*P* < 0.001] are indicated by an asterisk.)

**FIG 3:**
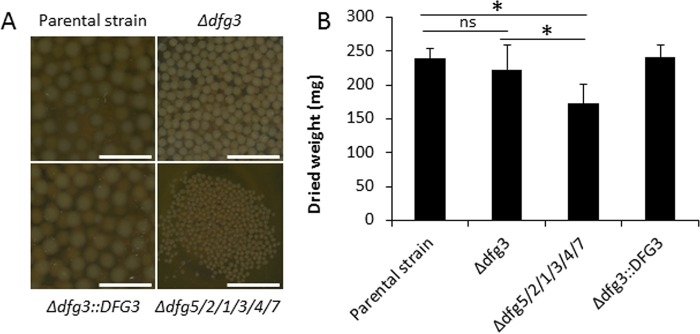
Morphology of the Δ*dfg* mutants in liquid medium. (A) Visual aspect of Δ*dfg* cultures in Sabouraud medium (10^6^ conidia/50 ml medium) after 24 h at 37°C (bar, 1 cm). (B) Mycelial dry weight of the Δ*dfg* mutants quantified after 24 h of growth in Sabouraud medium at 37°C (10^7^ conidia/50 ml medium). (Values represent means and standard deviations of results from four different experiments; statistically significant differences [*P* < 0.001] are indicated by an asterisk. ns, not significant.)

At 37°C, no growth differences were observed for the single-deletion strains (Δ*dfg1*, Δ*dfg2*, Δ*dfg4*, Δ*dfg5*, and Δ*dfg7*) and multiple-deletion strains (Δ*dfg5*/*2*, Δ*dfg5*/*2*/*1*, Δ*dfg5*/*2*/*1*/*4*) in comparison to the parental strain during growth on malt agar medium (Fig. 2A). Only the single-knockout Δ*dfg3* mutant and the multiple-knockout mutants containing a *DFG3* deletion (Δ*dfg5*/*2*/*1*/*3*, Δ*dfg5*/*2*/*1*/*3*/*4*, and Δ*dfg5*/*2*/*1*/*3*/*4*/*7*) displayed a significant reduction in vegetative mycelial growth compared to the parental strain and the *DFG3* revertant strain (Fig. 2). In the sextuple-deletion mutant, the absence of *DFG6* expression was verified by qRT-PCR at different stages of development of A. fumigatus (not shown). This result indicated that the sextuple-deletion mutant can be considered to be a mutant lacking the entire DFG gene family. Even though all mutants were evaluated, only the phenotypic analyses of the *Δdfg3* single-deletion mutant and the *Δdfg5*/*2*/*1*/*3*/*4*/*7* sextuple-deletion mutant are reported here. The differences in the amounts of mycelium produced by the Δ*dfg* mutants and the parental strain in Sabouraud liquid medium were much lower than those seen in agar media (Fig. 3B). However, the mycelia of these mutants grown in both solid and liquid media were highly branched (Fig. 2B). In liquid cultures, this hyperbranching phenotype resulted in the production of a myriad of very small and tight fungal balls which looked different from the homogeneous mycelial mass produced by the parental strain (Fig. 3A).

### Conidiation, conidial morphology of Δ*dfg* mutants, and susceptibility to drugs.

**FIG 4:**
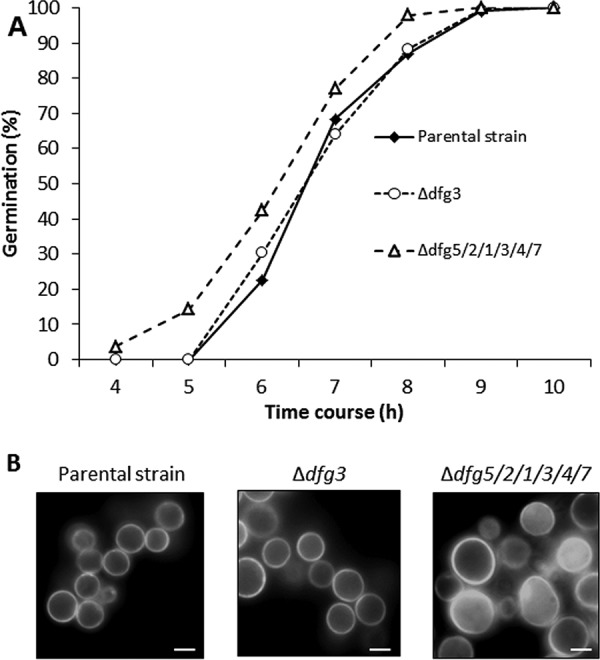
Conidial germination of the Δ*dfg* strains. (A) Germination kinetics of the parental and Δ*dfg* mutants. (B) Calcofluor white staining of swollen conidia of the parental strain and Δ*dfg* mutants (incubated in liquid Sabouraud medium for 4 h at 37°C). The estimated proportion (50%) of intracellularly fluorescent swollen conidia was determined by counting 200 conidia for the Δ*dfg5*/*2*/*1*/*3*/*4*/*7* mutant. (Bar, 2 μm.)

**FIG 5:**
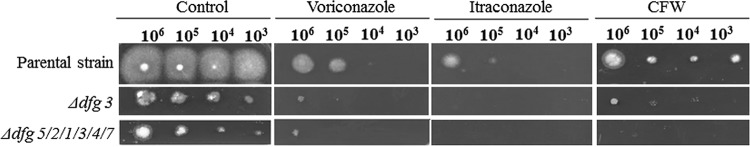
Sensitivity of the parental and Δ*dfg* strains to drugs. The levels of sensitivity of the Δ*dfg* mutants to calcofluor white (CFW [50 μg/ml]) and azole compounds (voriconazole [100 ng/ml] and itraconazole [50 ng/ml]) were determined after 72 to 96 h of growth at 37°C on RPMI agar medium.

The capacity of the different mutants to conidiate was assayed on malt agar-containing tubes following incubation for 10 days at room temperature. At 37°C, the slant was entirely covered by the fungus and no conidiation difference was observed for the single-deletion strains (Δ*dfg1*, Δ*dfg2*, Δ*dfg4*, Δ*dfg5*, and Δ*dfg7*) and multiple-deletion strains (Δ*dfg5*/*2*, Δ*dfg5*/*2*/*1*, and Δ*dfg5*/*2*/*1*/*4*) compared to the parental strain. Interestingly, the single-deletion *Δdfg3* mutant produced as many conidia as the parental strain whereas the sextuple-deletion Δ*dfg5*/*2*/*1*/*3*/*4*/*7* mutant displayed a drastic reduction in conidiation (Table 1). This conidiation defect could be compensated for by the addition of 6% KCl (Table 1). Similarly, 52% of the resting conidia from the sextuple-deletion Δ*dfg5*/*2*/*1*/*3*/*4*/*7* mutant were intracellularly stained with fluorescein isothiocyanate (FITC), whereas the resting conidia from the parental and *Δdfg3* strains were weakly stained by FITC at the cell surface (Fig. S3). Moreover, conidia of the sextuple-deletion Δ*dfg5*/*2*/*1*/*3*/*4*/*7* mutant germinated faster (∼18% germ tubes were formed after 4 h of incubation at 37°C) than those of the Δ*dfg3* and parental strains, which showed nearly no germinated conidia after the same 4 h of incubation time (Fig. 4A). While conidia of the parental and mutant strains were similar in size in the resting stage, the swollen conidia of the sextuple-deletion Δ*dfg5*/*2*/*1*/*3*/*4*/*7* mutant were approximately 1.5 times larger than those of the parental strain (Fig. 4B). In line with what was seen for FITC staining, calcofluor white (CFW) staining indicated that the cell walls of sextuple-deletion Δ*dfg5*/*2*/*1*/*3*/*4*/*7* mutant were more permeable than those of the parental strain, since intracellular labeling of around 50% of the swollen conidia was seen following 5 min of exposure to the stain (Fig. 4B). The single-deletion Δ*dfg3* mutant and the sextuple-deletion Δ*dfg5*/*2*/*1*/*3*/*4*/*7* mutant were more susceptible to voriconazole and itraconazole and to the cell wall-disturbing calcofluor white dye than the parental strain on RPMI medium after 72 h of incubation at 37°C (Fig. 5). All these conidial phenotypes suggested that the cell wall integrity was altered in the Δ*dfg5*/*2*/*1*/*3*/*4*/*7* mutant and that the alterations were associated with defects in cell wall permeability.

**TABLE 1:** Conidiation of the Δ*dfg* mutants and the parental strains in the presence or absence of KCl

Strain	No. of conidia grown in^*a*^:	
	Malt agar (2%)	Malt agar + KCl (6%)
Parental strain	(2.6 × 10^8^) ± 0.28	1.0 × 10^8^ ± 0.15
*Δdfg3* mutant	(2.4 × 10^8^) ± 0.10	ND
*Δdfg5*/*2*/*1*/*3*/*4*/*7* mutant	(2.1 × 10^5^) ± 0.68	4.4 × 10^7^ ± 0.87

a Data represent means and standard deviations (SD) of results from three different experiments. ND, not determined.

10.1128/mSphere.00397-19.4 FIG S3 FITC staining of resting conidia. The estimated proportion (52%) of intracellularly stained conidia was determined by counting 165 conidia for the Δ*dfg5*/*2*/*1*/*3*/*4*/*7* mutant. Bar, 100 μm. Download FIG S3, PPTX file, 1.77 MB. Copyright © 2019 Muszkieta et al. 2019 Muszkieta et al. This content is distributed under the terms of the Creative Commons Attribution 4.0 International license.

### Impact of DFG deletions on cell wall galactomannan (GM).

**FIG 6:**
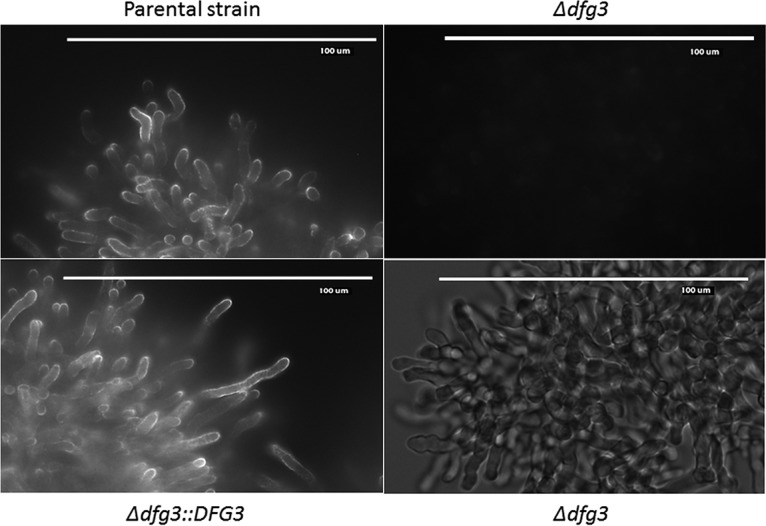
Labeling of the galactomannan present at the cell surface of the Δ*dfg* mutant complemented strain and the parental strain by an anti-Galf antibody. Mycelia grown for 16 h at 37°C in Sabouraud medium were fixed with *p*-formaldehyde and subjected to immunolabeling with anti-Galf antibody and anti-rat FITC secondary antibody. (Bar, 100 μm.) Both fluorescence and bright-field images are shown for the *Δdfg3* mutant.

**FIG 7:**
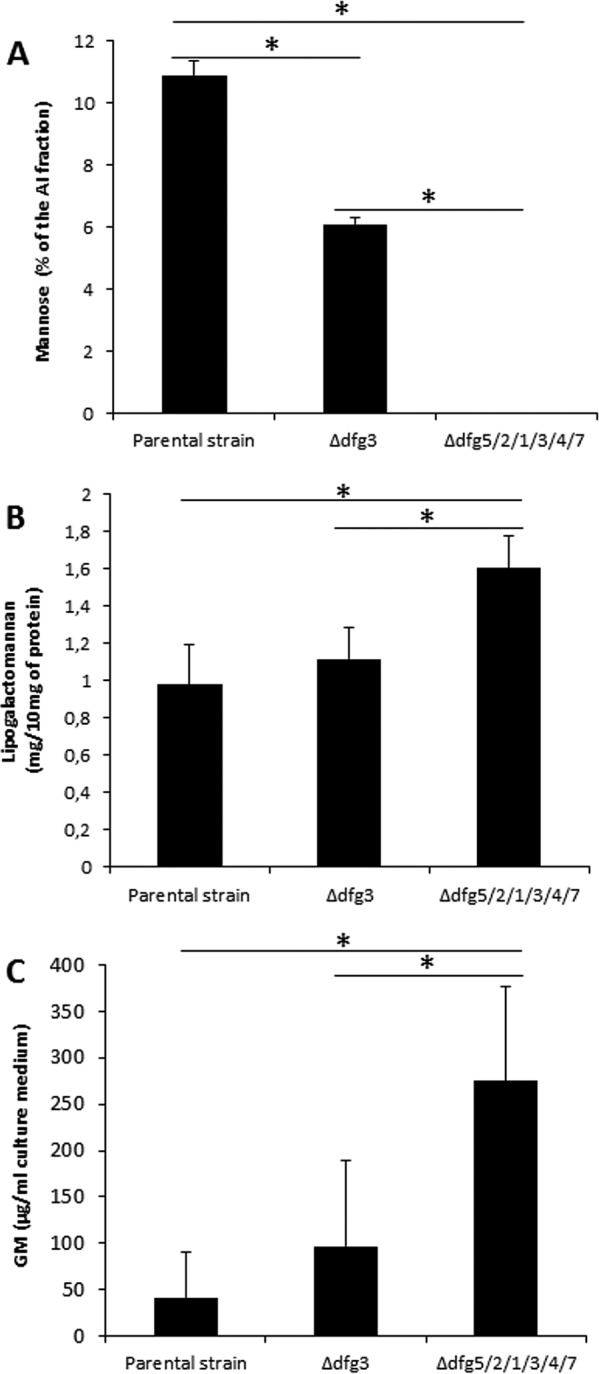
Impact of *DFG* deletion on galactomannan localization. (A) Galactomannan content in the alkali-insoluble fraction of the cell wall (estimated as the amount of mannose in the alkali-insoluble fraction). (B) Lipogalactomannan content extracted from membrane preparation. (C) Galactomannan levels estimated in the culture supernatant (quantified by sandwich enzyme-linked immunosorbent assay [ELISA] using a Platelia kit). Values represent means and standard deviations of data from three different experiments. Statistically significant differences (*P* < 0.05) are indicated by an asterisk.

In A. fumigatus, GM is either cross-linked to cell wall β-(1,3)-glucans or membrane bound through a GPI anchor (LGM) (14–16). The chemical compositions of the alkali-insoluble (AI) and alkali-soluble (AS) fractions of the A. fumigatus cell wall of parental and mutant strains are shown in Table 2. A decrease in the level of cell wall galactomannan (GM) was observed in both the AI and AS fractions in the *Δdfg3* mutant, and its complete absence was noted in the AI fraction of the Δ*dfg5*/*2*/*1*/*3*/*4*/*7* mutant. Quantification of GM carried out with the Δ*dfg* mutants was confirmed *in situ*, as shown by the absence of labeling of the GM with an antigalactofuran monoclonal antibody at the cell surface of the Δ*dfg3* mutant (Fig. 6). The reduction in the level of cell wall GM was primarily compensated for by an increase in the glucose content within the AI fraction and by an increase in the galactosamine content within the AS fraction, corresponding to larger amounts of β-(1,3)-glucan and galactosaminogalactan, respectively (Table 2). The single-deletion Δ*dfg3* mutant showed a significant reduction in GM content (40% of the level seen with the AI fraction) compared with the parental strain, whereas no cell wall cross-linked GM was detected in the sextuple-deletion mutant (Fig. 7A). In addition, quantification of the GM in the single-deletion *Δdfg3* mutant and the multiple-deletion *Δdfg5*/*2*/*1*/*3*, *Δdfg5*/*2*/*1*/*3*/*4*, and *Δdfg5*/*2*/*1*/*3*/*4*/*7* mutants showed an additive effect consisting of a concomitant decrease in the level of cell wall GM content corresponding to an increase of the number of *DFG* deletions.

**TABLE 2:** Monosaccharide compositions of cell wall fractions from the parental and Δ*dfg* strains^*a*^

Strain	% indicated monosaccharide in:								AI (%)
	Alkali-insoluble (AI) fraction				Alkali-soluble (AS) fraction				
	Man	Glc	Gal	GlcN	Man	Glc	Gal	GalN	
Parental strain	10.90 ± 0.35	46.25 ± 0.75	11.26 ± 0.09	31.07 ± 1.11	4.78 ± 1.79	74.42 ± 1.61	10.67 ± 0.04	9.67 ± 1.20	68.91 ± 4.24
Δ*dfg3*	6.10 ± 0.17	50.17 ± 0.65	10.43 ± 0.23	32.10 ± 0.82	2.18 ± 0.13	55.81 ± 1.98	8.79 ± 1.10	32.78 ± 3.07	58.61 ± 4.67
Δ*dfg5*/*2*/*1*/*3*/*4*/*7*	0	61.56 ± 2.11	0	34.70 ± 1.62	0.47 ± 0.09	61.47 ± 4.01	1.85 ± 1.08	35.61 ± 4.99	56.46 ± 8.56

a Data represent means and standard deviations (SD) of results from three different experiments. Man, mannose; Glc, d-glucopyranose; Gal, d-galactopyranose; GlcN, 2-amino-2-deoxy-d-glucopyranose.

In contrast to the cell wall GM, the GM content was slightly increased in membrane preparations (Fig. 7B). The amount of GM in the *Δdfg3* mutant was close to 110% of the GM content in the membrane of the parental strain and reached 160% of the parental strain level in the sextuple-deletion mutant (Fig. 7B), In addition, no significant differences were observed in the molar ratios of the hexose content of the membrane-bound GM of the parental and the sextuple-deletion Δ*dfg5*/*2*/*1*/*3*/*4*/*7* strains. Structural analysis of membrane-bound GM showed the presence of terminal galactofuranose, 5-O-substituted galactofuranose, 2-O-substituted mannose, 6-O-substituted mannose, 2,6-di-O-substituted mannose, and 2,3-di-O-substituted mannose residues, typical of the GM sequence (14, 16). The lipid anchor of LGM was isolated from the sextuple-deletion mutant and analyzed by electrospray-mass spectrometry (ES-MS). The major pseudomolecular ion [M-H]^-^ at *m*/*z* = 1,086 characterized the GPI-related anchor of LGM with the presence of a glucosamine residue linked to an inositolphosphoceramide moiety, which was composed of a C_18_-phytosphingosine and a 2-hydroxy-C_24:0_ fatty acid, as previously described (16) (Fig. S4). Taking into account all these structural data, the level of GM from the Δ*dfg5*/*2*/*1*/*3*/*4*/*7* mutant is identical to that from the parental strain, indicating that the intracellular biosynthetic pathway of GM was not altered by *DFG* deletions.

10.1128/mSphere.00397-19.5 FIG S4 Negative-ion ES-MS spectra of the butanol-soluble products released by mild HCl hydrolysis (50 mM HCl for 15 h at 100°C) of the purified lipogalactomannan produced by the Δ*dfg5*/*2*/*1*/*3*/*4*/*7* mutant. As previously described for LGM of the wild-type strain of A. fumigatus (16), the ES-MS spectra revealed one main pseudomolecular ion at *m*/*z *=* *1,085.714 corresponding to a glucosamine linked to a inositolphosphoceramide (GlcN-IPC) where the ceramide is composed of a C_18_-phytophygosine and a 2-hydroxy-C_24:0_ fatty acid. Ions at *m*/*z* = 1,069.713 and 1,099.719 were due to the absence of a hydroxyl group or the addition of a carbon to the fatty acid chain. Ion masses at *m*/*z* = 1,247.712 and 1,409.806, corresponding to increases of 162 and 324 compared to the main ion (*m*/*z* = 1,085.714), respectively, were due to the presence of one or two mannose residues linked to the glucosamine-IPC anchor. Download FIG S4, PDF file, 0.03 MB. Copyright © 2019 Muszkieta et al. 2019 Muszkieta et al. This content is distributed under the terms of the Creative Commons Attribution 4.0 International license.

Interestingly, the amount of GM in the culture filtrate of the Δ*dfg3* and Δ*dfg5*/*2*/*1*/*3*/*4*/*7* mutants increased substantially in comparison to the level seen with the parental strain (Fig. 7C). An additive effect with a concomitant increase in the free GM content in the culture filtrate was seen with the increase of the number of *DFG* deletions.

In conclusion, the intracellular biosynthetic pathway of GM was not altered by *DFG* deletions qualitatively or quantitatively. In contrast, the *DFG* deletion resulted in a defect in insertion of this polysaccharide into the cell wall and in associated extracellular release of the GM into the external medium. Even though only the deletion of the *DFG 3* gene resulted in a morphogenetic phenotype among all the single-deletion Δ*dfg* mutants, the comparison of the GM phenotypes of the Δ*dfg3* mutant and the Δ*dfg5*/*2*/*1*/*3*/*4*/*7* multiple-deletion mutant indicated additivity of the functions of these DFG proteins in the insertion of GM into the cell wall.

### Complementation of the *Δdfg5*/*dcw1* yeast mutant by *AfDFG3*.

**FIG 8:**
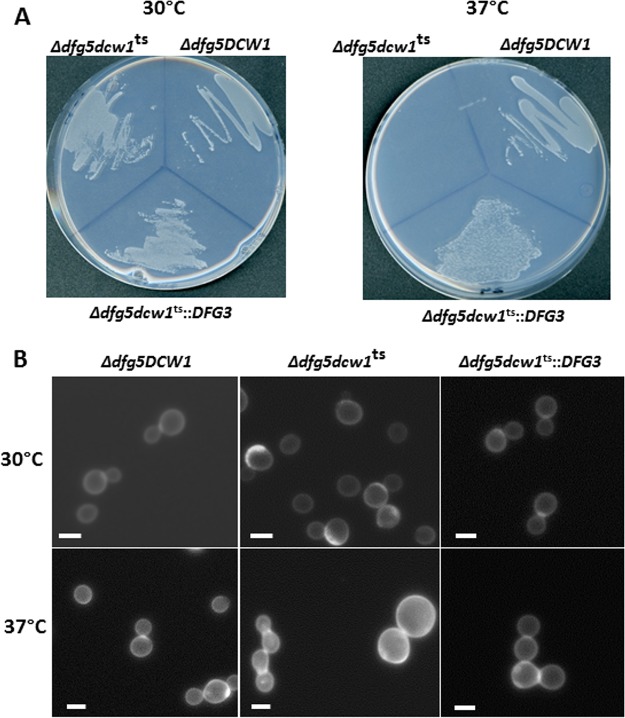
Functional complementation of yeast *dcw1^ts^*/*Δdfg5* mutant by Af*DFG3*. (A) Growth on YNB plate medium after 3 days at 30°C and 37°C. (B) Calcofluor white staining of the cells after 2 days of culture in YNB liquid medium at 30°C and 37°C. An average of 15% of enlarged yeast cell (>15 μm) were estimated in the *dcw1ts*/∆*dfg5* mutant by counting 400 cells (bar, 6 μm).

A double-knockout *Δdcw1*/*Δdfg5* mutant was shown previously to be synthetically lethal in yeast (11). Functional complementation by A. fumigatus *DFG3* (*AfDFG3*) in S. cerevisiae was investigated using a thermosensitive *dcw1^ts^*/*Δdfg5* mutant. This mutant was unable to grow on solid minimum medium at 37°C and produced a 15% proportion of enlarged yeast cells in liquid medium at 37°C. The introduction of the *AfDFG3* gene into the mutant strain restored normal growth at 37°C at a level that was similar to that seen with the control *DCW1*/*Δdfg5* mutant strain (Fig. 8), showing that the *AfDFG3* genes and S. cerevisiae *DCW1* (*ScDCW1*) genes share similar biological activities.

## DISCUSSION

### The phenotypes of *Δdfg* mutants are different among yeast and mold.

In this report, we describe the results of characterization of the *DFG* family in A. fumigatus. The deletion of *DFG* genes led to a major reduction in vegetative growth with hyperbranched hyphae. In addition, it was shown that the genes of the *DFG* family have additive biological activities, as shown also in S. cerevisiae. Nine *DFG* paralogs have been annotated in the genome of Neurospora crassa, and among the single-deletion mutants, only two have been studied, both of which showed altered morphology (17). Of the two mutants, the *Δdfg5* mutant (NCU03770) showed a reduction in colony size and a hyperbranched mycelial pattern, in similarity to the *Δdfg3* mutant in A. fumigatus. The *Δdcw1* mutant (NCU08127) did not show a significant growth phenotype in *Neurospora*, but the growth phenotype of the *Δdfg5*/*Δdcw1* double-deletion mutant of N. crassa showed more-severe effects than were seen with the strain with a single *Δdfg5* deletion, with increased susceptibility to cell wall-perturbing agents, such as caspofungin (17). In yeast, single deletion of the paralogous *DFG5* gene or *DCW1* gene also leads to cell wall alterations and growth defects. The Sc*Δdfg5* mutant has been reported previously to be defective in filamentous growth, cell polarity, and elongation (18). Mutant Sc*Δdcw1* (for “defective in cell wall”) was hypersensitive to zymolyase, a cell wall-digesting enzyme, suggesting a role in cell wall organization (19). In C. albicans, a mutant lacking *DFG5* was defective in hyphal formation at alkaline pH whereas a mutant lacking *DCW1* had no obvious phenotype. In both C. albicans and S. cerevisiae, deletions of *DFG5* and *DCW1* were synthetically lethal (12, 19). In A. fumigatus, the entire deletion of all members of the DFG family (since DFG6 remained unexpressed in the sextuple-deletion mutant) was not lethal. Our data showed, as often reported for orthologous genes of yeast and filamentous fungi, that the phenotypes of their respective mutants were different. Among the single-deletion mutants, only DFG3 deletion led to a growth defect; the major phenotypic difference was seen in the sextuple-deletion mutant, where additive effects on mycelial morphology, germination rate, drug sensitivity, cell wall composition, and permeability were observed with increasing numbers of DFG gene deletions.

### Function of the Dfg proteins.

On the basis of sequence homology, Dfg proteins have been assigned to the GH76 glycoside hydrolase family in the CAZy database (20). Dfg proteins were predicted to act as endo-α-(1,6)-mannanases based on the homologies of the sequence of a single member of this family with that of the *Aman6* protein from Bacillus circulans (21, 22). However, to date, there has been no biochemical evidence found to indicate that any fungal Dfg protein acts as an endo-α-(1,6)-mannanase or possesses any hydrolytic activity with respect to cell wall polysaccharides. In our hands, efforts to produce recombinant AfDfg3p were not successful. Since *Sc*Dcw1p showed 32% identity with *Af*Dfg3p (see Table S1 in the supplemental material) and the S. cerevisiae *dcw1^ts^*/*Δdfg5* mutant was functionally complemented by *Af*DFG3 (Fig. 8), we investigated the enzymatic activity of the recombinant *Sc*Dcw1 protein. The glycosylhydrolase activity of the recombinant *Sc*Dcw1 protein was assayed with several α-mannosides (see Text S1 in the supplemental material). The transglycosylase activity has been also investigated using galactomannan as a donor and soluble β-(1,3)-glucans or the cell wall AI fraction from S. cerevisiae as an acceptor (Text S1). No hydrolase or transfer activity has been detected with any of these substrates, even with the sensitive fluorometry assay used with the bacterial GH76 member (23) (Text S1). The enzyme activity of fungal GH76 family members therefore remains uncharacterized but certainly does not represent that of an α-mannanase.

10.1128/mSphere.00397-19.1 TEXT S1 Supplemental methods. Production of recombinant ScDcw1p and enzymatic assays. Download Text S1, DOCX file, 0.09 MB. Copyright © 2019 Muszkieta et al. 2019 Muszkieta et al. This content is distributed under the terms of the Creative Commons Attribution 4.0 International license.

The increased secretion of GPI-anchored proteins in the *Δdfg5* mutant or the *Δdcw1* mutant in S. cerevisiae suggested that these proteins were involved in the cross-linking of GPI-CWPs to the cell wall glucans (17, 24). However, in addition to the absence of any biochemical demonstration of such transglycosylase activity, ascribing a cross-linking function remains controversial. In C. albicans, shutoff strains for *DFG5* and *DCW1* were previously shown to exhibit the release of both GPI-anchored and non-GPI cell wall mannoproteins (25). Moreover, in previous studies, secretion of very few GPI proteins, including Acw1p (=ECM33), Gel1p in N. crassa, and Cwp1p in S. cerevisiae (11, 17), was demonstrated, whereas localization of the major GPI-anchored protein, Gas1p, remained unchanged in both *Dfg5* and *Dcw1* single-deletion mutants in S. cerevisiae (26). In N. crassa, the same proteins were released in the culture supernatant of mutants with deletions of very different genes, such as those encoding a putative hyaluronic synthase (*CPS1*) or mannosyltransferase (*OCH1*) (27, 28). In A. fumigatus, *DFG* deletions led also to a 3-fold increase in the amount of secreted proteins (see Fig. S5 in the supplemental material). However, the six overproduced proteins were mainly non-GPI-anchored proteins such as endopolygalacturonase, endochitinases, and pectate lyase (Fig. S5). Such a modification of the secreted protein pattern has been indeed reported in other A. fumigatus mutants after genes coding for proteins with a known function in cell wall biosynthesis were deleted (29, 30). Taking the data together, the results suggest that Dfg proteins are not involved in the cell wall localization of GPI-anchored proteins, at least in filamentous fungi. The modification of the cell wall structure due to the loss of cell wall galactomannan in the *Δdfg3* single-deletion and multiple-deletion mutants leads to facilitation of the release of secreted proteins in transit through the cell wall. These cell wall modifications lead to a change in permeability such as was demonstrated when the Δ*dfg3* single-deletion mutant and the Δ*dfg5*/*2*/*1*/*3*/*4*/*7* multiple-deletion mutant were incubated with CFW or FITC (Fig. 5; see also Fig. S3). Intracellular labeling with FITC and CFW has been repeatedly performed with cell wall mutants of A. fumigatus to demonstrate the effect of a gene deletion on cell wall permeability (5, 8, 29–32). However, we have no idea of the nature of the material stained intracellularly by CFW or FITC.

10.1128/mSphere.00397-19.6 FIG S5 Results of 12% SDS-PAGE of secreted proteins from 24-h liquid culture maintained at 37°C in Sabouraud medium. Thirty-microgram volumes of proteins were loaded. Proteins were stained with Coomassie blue and identified after trypsin digestion and MS/MS analysis. Download FIG S5, PDF file, 0.5 MB. Copyright © 2019 Muszkieta et al. 2019 Muszkieta et al. This content is distributed under the terms of the Creative Commons Attribution 4.0 International license.

### Dfg proteins and the cell wall GM in A. fumigatus.

GM is a polymer composed of a linear α-(1,2)/α-(1,6)-mannan chain with short side chains of β-(1,5)-galactofuran (Galf) (33). This polysaccharide can be covalently bound to β-(1,3)-glucans in the cell wall, bound to the plasma membrane by a GPI anchor, or present in the extracellular environment. Despite of its importance in fungal morphogenesis and in the host immune response (31), GM biosynthesis is poorly understood. Previous studies have shown that GM biosynthesis takes place in the Golgi apparatus, into which sugar-donor (UDP-galactofuranose and GDP-mannose) are transported prior to polysaccharide polymerization (34–36). The polymerization of galactofuran was due to the action of a specific galactofuranosyltransferase, GfsA (37, 38). Two Ktr/Mnn2 mannosyltransferases have been recently identified to be essential to the mannan polymerization (31, 39). Interestingly, the absence of Ktr mannosyltransferases led to the absence of cell wall GM and to filamentous growth with an hyperbranched mycelium and a conidiation defect (31). The absence of cell wall cross-linked GM in Δ*dfg* mutants could suggest a putative role of *Dfg* family members in mannan polymerization. However, our biochemical characterization showed that the *dfg* mutants still produced membrane-bound GM with the same chemical structure as that of the wild type (WT), showing that Dfg proteins are not involved in GM biosynthesis. Our study results show that these *DFG* proteins play a key role in the cross-linking of GM to β-(1,3)-glucans through an as-yet-undiscovered form of remodelling activity. The study also confirmed that all of the polysaccharide components of the cell wall skeleton [β-(1,3)-glucans, chitin, and galactomannan] are essential for fungal life whereas the alkali amorphous content has a nonstructural function and is mainly involved in the communications with the external milieu (32).

## MATERIALS AND METHODS

### Culture conditions.

Parental and mutant strains of A. fumigatus were grown at 37°C in *Aspergillus* minimal medium (AMM; 1% glucose and 5 mM ammonium tartrate), Sabouraud medium (2% glucose, 1% Mycopeptone [Difco]), RPMI medium (Sigma), or 2% malt medium (Cristomalt). Media were either used in liquid form or supplemented with 2% agar. When necessary, 6% KCl was added to the media to enhance conidiation. Conidia were collected from agar medium plates after 10 days of growth at 37°C, using water containing 0.05% Tween 20.

### Construction and complementation of the Δ*dfg* mutants.

The single-deletion and multiple-deletion mutants were constructed in the CEA17_Δ*akuB*^KU80^ background (40) using the *β*-rec/six site-specific recombination system (13). The self-excising *β*-rec/six blaster cassette containing the hygromycin resistance marker was released from pSK529 plasmid using FspI restriction enzyme. The *dfg* replacement cassette containing the marker module flanked by 5′ and 3′ homologous regions of the target gene was generated by using primers listed in Table S3 in the supplemental material and cloned into the pUC19 vector using GeneArt seamless cloning and assembly (Life Technologies, Carlsbad, CA, USA). The corresponding replacement cassettes were released from the resulting vector via the use of either EcoRV or FspI, respectively. The CEA17Δ*akuB*^KU80^ parental strain was transformed with the *dfg* replacement cassettes by electroporation to generate the *dfg* single-deletion mutants. Transformants were analyzed by PCR and Southern blotting using the digoxigenin (DIG) probe protocol (Roche Diagnostics) (see Fig. S2 in the supplemental material). For the construction of multiple-deletion strains, single-deletion mutants were cultivated in the presence of 2% xylose-containing minimal medium, which allows the excision of the selection marker by triggering recombination between the six recognition regions in the β-rec/six cassette. Because deletion of *DFG4* results in resistance to hygromycin, the sextuple-deletion mutant was constructed with the phleomycin resistance marker (41). The deletion cassette was made by fusion PCR with primers listed in Table S3 by using the double-joint PCR method.

10.1128/mSphere.00397-19.9 TABLE S3 Primer list used in this study. Download Table S3, DOCX file, 0.02 MB. Copyright © 2019 Muszkieta et al. 2019 Muszkieta et al. This content is distributed under the terms of the Creative Commons Attribution 4.0 International license.

Complementation of the Δ*dfg3* mutant was carried out by reintroduction of the parental copy of the gene flanked by the hygromycin resistance cassette and a 3′ flanking region (Fig. S2; see also Table S3). The complementation cassette was transformed into the cassette-excised Δ*dfg3* mutant. The presence of the parental copy of the gene at the *DFG3* locus was confirmed by Southern blot analysis (Fig. S2). All mutants are detailed in Table S2.

### Complementation of *ScΔdcw1^ts^*/*Δdfg5* mutant by *AfDFG3*.

S. cerevisiae BY230 strain was a derivative of S288c in which both *DCW1* and *DFG5* chromosomal loci were deleted. Since they are synthetically lethal, the strain therefore also carried p413TEF.*DCW1*, a *URA3* plasmid (42) containing the *DCW1* gene. The *DCW1* deletion in a Δ*dfg5* strain was obtained from the haploid BY4741 deletion collection. A *DCW1* deletion plasmid was generated by cloning 5′ and 3′ flanking regions of *DCW1* into YIPLAC211 (43). The flanking regions were generated by PCR with primers 1975, 1976, 1977, and 1978 (Table S3). Following digestion with Mlu1, this plasmid was integrated at the *DCW1* locus of the *Δdfg5* strain. This integrant was transformed with a CEN-ARS *HIS3* plasmid, p413TEF.*DCW1*, carrying the *DCW1* open reading frame (ORF) cloned as a BamH1-Xho1 fragment (generated with primers 2309 and 2310 and sequence verified). The genomic locus was then deleted by selection on plates with 5-fluoro-orotic acid followed by screening for the ORF deletion by PCR using flanking primers 2306 and 2307 to generate strain JT347 (*DCW1*/*Δdfg5).* In strain JT346 (*dcw1^ts^*/*dfg5Δ*), plasmid shuffling was used to replace the p413TEF.*DCW1* plasmid with p413TEF.*dcw1^ts^*, a temperature-sensitive allele unable to support growth at 37°C (J. Trow and B. P. Cormack, unpublished data). Yeast strain JT346 (*dcw1^ts^*/*Δdfg5*) was used for complementation performed with Af*DFG3*. Yeasts were grown at 30°C and 220 rpm in either a standard YEPD medium (10 g/liter yeast extract, 20 g/liter Bacto peptone, 20 g/liter glucose) or in YNB medium (1.7 g/liter yeast nitrogen base without amino acids and ammonium, 5 g/liter ammonium sulfate, 20 g/liter glucose) supplemented with the auxotrophic requirements.

The Af*DFG3* cDNA was synthetized by Life Technology SAS (St Aubin, France). A NotI restriction site and an XhoI restriction site were incorporated at the 5′ and 3′ ends of the cDNA, respectively, and cloned into pMA plasmid containing an ampicillin resistance marker. The Af*DFG3* cDNA was subcloned in plasmid pREP3-ADH (44) containing a *LEU2* gene to obtain pREP3-Af*DFG3* after digestion by NotI/XhoI. Then, the *dcw1^ts^*/*Δdfg5* mutant was transformed with 3 μg of plasmid pREP23-Af*DFG3* following the lithium acetate method (45). As a control, the *dcw1^ts^*/*Δdfg5* mutant was transformed with the pREP3-ADH plasmid alone. The transformants were selected on YNB plates without leucine, and the levels of expression of the Af*DFG3* genes in the S. cerevisiae mutant were checked by RT-PCR using primer pair Af*DFG3*comp1 and Af*DFG3*comp2 (Table S3). Phenotypes of the JT347 (*DCW1*/*Δdfg5*), JT346 (*dcw1^ts^*/*Δdfg5*), and complemented (*dcw1^ts^*/*Δdfg5*::*AfDFG3*) strains were analyzed on YNB medium at 30°C and 37°C.

### Quantitative real-time PCR analysis.

Fungal material was disrupted by the use of 0.5-mm-diameter glass beads in 500 μl of saturated phenol (Interchim, Montluçon, France) (pH 4.5), and RNA was isolated as described earlier (5) or by using a Qiagen RNeasy minikit. Quantitative PCR assays were performed as previously described (5). The expression ratios were normalized to *EF1α* expression levels and were calculated according to the 2^Δ^*^CT^* (threshold cycle) method (46). The absence of genomic DNA contamination was verified with negative controls without reverse transcriptase. Three independent biological replicates were performed. The specificity of each primer (Table S3) was checked by agarose gel electrophoresis of RT-PCR products.

### Phenotype of the Δ*dfg* strains.

Mycelial growth of the different strains was measured on 2% malt solid media after 48 h of incubation at 37°C. Agar pieces containing the entire colony of each of the strains were boiled 5 min in water to eliminate medium and extensively washed with hot water, and mycelial dry weight was recorded after overnight incubation at 80°C. The morphology of the mutant strains was determined during a kinetic study of the fungus grown in Sabouraud medium after staining of the fungus with calcofluor white (final concentration of 5 μg/ml). Conidia and mycelium were observed with a fluorescence microscope (Evos; Life Technologies) (excitation delta [λ ex], 357/44 nm; emission delta [λem], 447/66 nm). To quantify growth, 24-h-old mycelial cultures that had been maintained at 37°C were filtered, washed, and dried overnight at 80°C and the dry weights were recorded. The conidiation rates were estimated following inoculation of conidial suspensions (100 μl, 10^4^/ml) into three tubes containing 2% malt agar (10 ml/tube) or malt agar containing 6% KCl. After 10 days at 25°C, conidia were recovered with aqueous 0.05% Tween 20 solution and counted using a hemocytometer.

The conidial permeability determinations were followed in the presence of FITC (47). Conidia were observed with a fluorescence microscope (Evos; Life Technologies) (λex, 470/22 nm; λem, 510/42 nm).

The susceptibility of strains to antifungal and cell wall-disturbing compounds was estimated by spotting 10-fold serial dilutions of conidia (starting from 2 × 10^6^ spores) onto RPMI plates supplemented with the following drugs: itraconazole [50 ng/ml], voriconazole [100 ng/ml], amphotericin B [250 ng/ml], caspofungin [75 ng/ml] and calcofluor white [50 μg/ml]. Plates were incubated for 72 h at 37°C in a humid atmosphere.

### Carbohydrate analysis of the cell wall, membrane fractions, and culture supernatant.

Following 24 h of growth in Sabouraud liquid medium at 37°C and 150 rpm, mycelia and culture supernatants were separated by filtration. Macromolecules from the culture medium were precipitated by the use of three volumes of ethanol at 4°C overnight and collected by centrifugation (5 min, 4,000 × *g*). Cell walls and membranes were obtained after mycelium disruption and centrifugation (5, 16). Lipogalactomannan (LGM) was purified from each membrane preparation as previously described (16). The amount of galactomannan present in the culture filtrate was estimated using a Platelia kit as described by the manufacturer (Bio-Rad, Marnes la Coquette, France) with purified GM as the standard. Prior to the GM assay, proteins from the culture supernatant were eliminated by solid-phase extraction (SPE) on Sep-Pak classic C_18_ cartridges (Waters) as previously described (48). Polysaccharides from the cell wall were separated based on their alkali solubility (5). Neutral hexoses and osamines were quantified by colorimetric and chromatographic assays (5, 49, 50). Proteins were quantified by use of the bicinchoninic acid (BCA) assay (Thermo Scientific), analyzed by SDS-PAGE, and identified by tandem mass spectrometry (MS/MS) analysis.

### Galactomannan analysis.

The analysis of glycosidic linkages in LGM was performed by methylation (51). The lipid anchor of LGM was released by mild acid hydrolysis (50 mM HCl, 100°C for 15 h), purified on a silica gel column, and analyzed by electrospray-mass spectrometry (16). Galactomannan (GM) amounts were estimated using a Platelia kit according to the instructions of the manufacturer (Bio-Rad, Marnes la Coquette, France) with purified GM as the standard. GM from the membrane preparation was solubilized with 2% Triton X-100. After trypsin digestion (1 mg trypsin for 10 mg of protein at 37°C for 24 h), GM was purified by SPE on Sep Pak classic C_18_ cartridges and eluted with 5% propanol-1 (48). Mass spectrometry analysis of the lipid anchor was carried out on a Synapt G2Si instrument (Waters Corp., Milford, MA, USA). The source temperature was set to 80°C. The capillary and cone voltages were set to 1,500 and 40 V. Time of flight (TOF) data were collected between *m*/*z* 50 and 2,000, at a low level of collision energy (10 eV), in negative mode. Argon was used as the collision gas. Scans were collected for 1 s. An external calibration was done with clusters of NaI, and the mass range of calibration was *m*/*z* 50 to 2,000. Mass Lynx 4.1 was used for both acquisition and data processing. Samples were dissolved in chloroform/methanol (1/4 [vol/vol]) and introduced in nanoelectrospray mode via the use of a coated, medium-sized nano-electrospray ionization (ESI) capillary (Proxeon).

### Fluorescence microscopy.

Mycelia of the Δ*dfg* and parental strains were fixed using *p*-formaldehyde (at a concentration of 2.5% in phosphate-buffered saline [PBS]) for one night at 4°C, washed three times with 0.1 M NH_4_Cl–PBS and once with PBS, and then incubated with antigalactomannan antibody (52). Galactomannan was labeled with a rat anti-galactofuran monoclonal antibody (EBA2; a kind gift of M. Tabouret, Bio-Rad, Steenvorde) and a secondary FITC-conjugated goat anti-rat (Sigma) antibody. A monoclonal antibody with the same isotype was used as a negative control.

### Statistical analysis.

At least three biological replicates were performed per experiment; the statistical significance of the results was evaluated by one-way variance analysis using JMP1 software (SAS Institute, Cary, NC, USA).
