# Supplementary material for: The Glycosylphosphatidylinositol-Anchored DFG Family Is Essential for the Insertion of Galactomannan into the β-(1,3)-Glucan–Chitin Core of the Cell Wall of Aspergillus fumigatus
Source: mSphere. 2019 Jul 31;4(4):e00397-19. doi: 10.1128/mSphere.00397-19 (PMC6669337; doi:10.1128/mSphere.00397-19)
Supplement: FIG S1 [file mSphere.00397-19-sf001.ppt]

## Slide 1
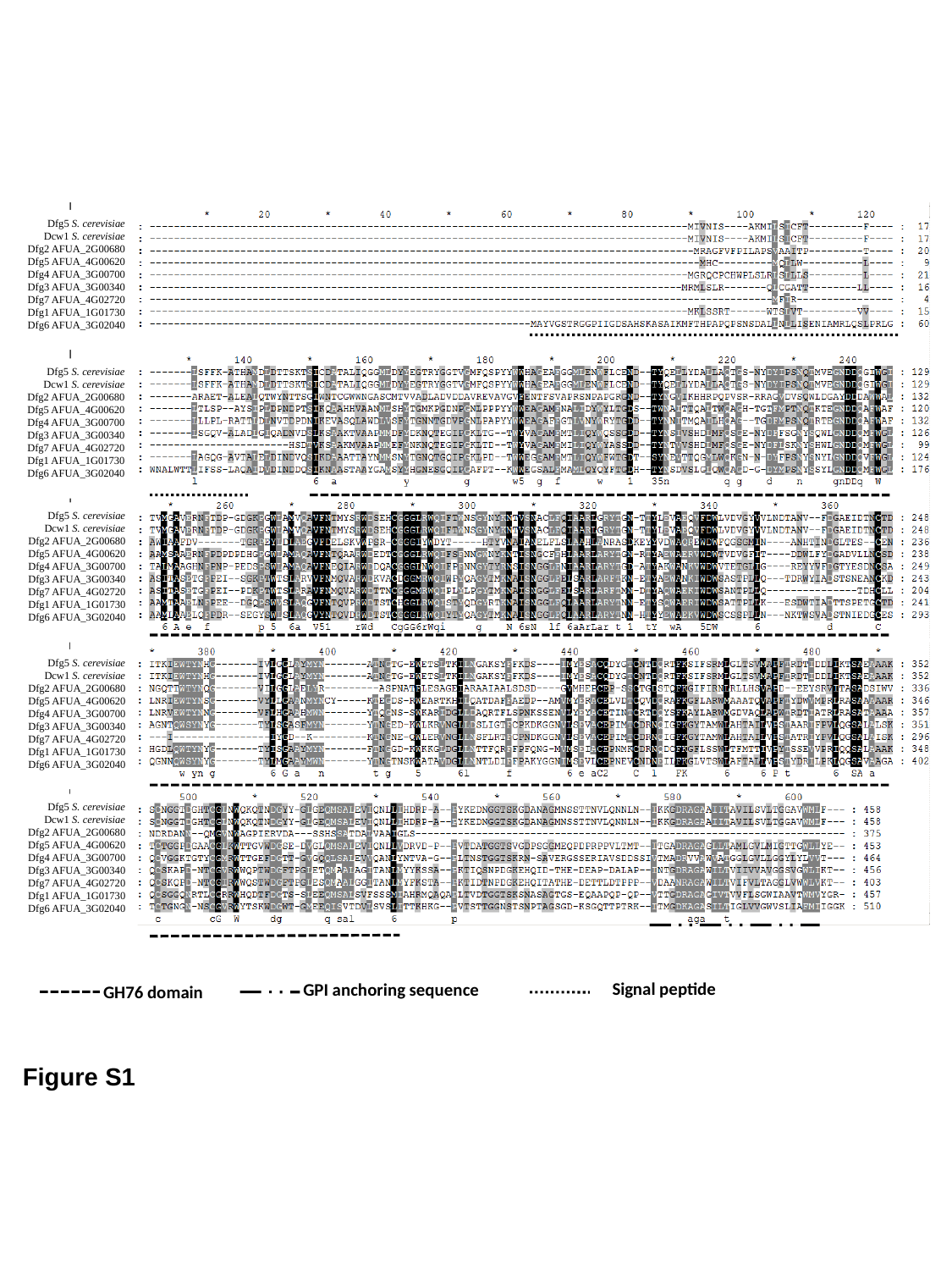

Dfg5 S. cerevisiae
Dcw1 S. cerevisiae
Dfg2 AFUA_2G00680
Dfg5 AFUA_4G00620
Dfg4 AFUA_3G00700
Dfg3 AFUA_3G00340
Dfg7 AFUA_4G02720
Dfg1 AFUA_1G01730
Dfg6 AFUA_3G02040
Dfg5 S. cerevisiae
Dcw1 S. cerevisiae
Dfg2 AFUA_2G00680
Dfg5 AFUA_4G00620
Dfg4 AFUA_3G00700
Dfg3 AFUA_3G00340
Dfg7 AFUA_4G02720
Dfg1 AFUA_1G01730
Dfg6 AFUA_3G02040
Dfg5 S. cerevisiae
Dcw1 S. cerevisiae
Dfg2 AFUA_2G00680
Dfg5 AFUA_4G00620
Dfg4 AFUA_3G00700
Dfg3 AFUA_3G00340
Dfg7 AFUA_4G02720
Dfg1 AFUA_1G01730
Dfg6 AFUA_3G02040
Dfg5 S. cerevisiae
Dcw1 S. cerevisiae
Dfg2 AFUA_2G00680
Dfg5 AFUA_4G00620
Dfg4 AFUA_3G00700
Dfg3 AFUA_3G00340
Dfg7 AFUA_4G02720
Dfg1 AFUA_1G01730
Dfg6 AFUA_3G02040
Dfg5 S. cerevisiae
Dcw1 S. cerevisiae
Dfg2 AFUA_2G00680
Dfg5 AFUA_4G00620
Dfg4 AFUA_3G00700
Dfg3 AFUA_3G00340
Dfg7 AFUA_4G02720
Dfg1 AFUA_1G01730
Dfg6 AFUA_3G02040
Signal peptide
GPI anchoring sequence
GH76 domain
Figure S1
