# Supplementary material for: The Glycosylphosphatidylinositol-Anchored DFG Family Is Essential for the Insertion of Galactomannan into the β-(1,3)-Glucan–Chitin Core of the Cell Wall of Aspergillus fumigatus
Source: mSphere. 2019 Jul 31;4(4):e00397-19. doi: 10.1128/mSphere.00397-19 (PMC6669337; doi:10.1128/mSphere.00397-19)
Supplement: TABLE S1 [file mSphere.00397-19-st001.docx]

**Table S1: Comparison of the *DFG* gene families in *S.cerevisiae* and *A.fumigatus*.**

|  |  | |
| --- | --- | --- |
|  | Dfg5p/Dcw1p (*S. cerevisiae*) | |
| Gene Name | Identity (%) | Similarity (%) |
| Dfg1p (AFUA_1G01730) | 35/37 | 51/53 |
| Dfg2p (AFUA_2G00680) | 15/16 | 30/30 |
| Dfg3p (AFUA_3G00340) | 29/32 | 47/49 |
| Dfg4p (AFUA_3G00700) | 37/39 | 55/58 |
| Dfg5p (AFUA_4G00620) | 42/42 | 58/60 |
| Dfg6p (AFUA_3G02040) | 30/33 | 44/49 |
| Dfg7p (AFUA_4G02720) | 25/28 | 42/44 |
