# Supplementary material for: The Glycosylphosphatidylinositol-Anchored DFG Family Is Essential for the Insertion of Galactomannan into the β-(1,3)-Glucan–Chitin Core of the Cell Wall of Aspergillus fumigatus
Source: mSphere. 2019 Jul 31;4(4):e00397-19. doi: 10.1128/mSphere.00397-19 (PMC6669337; doi:10.1128/mSphere.00397-19)
Supplement: FIG S2 [file mSphere.00397-19-sf002.ppt]

## Slide 1
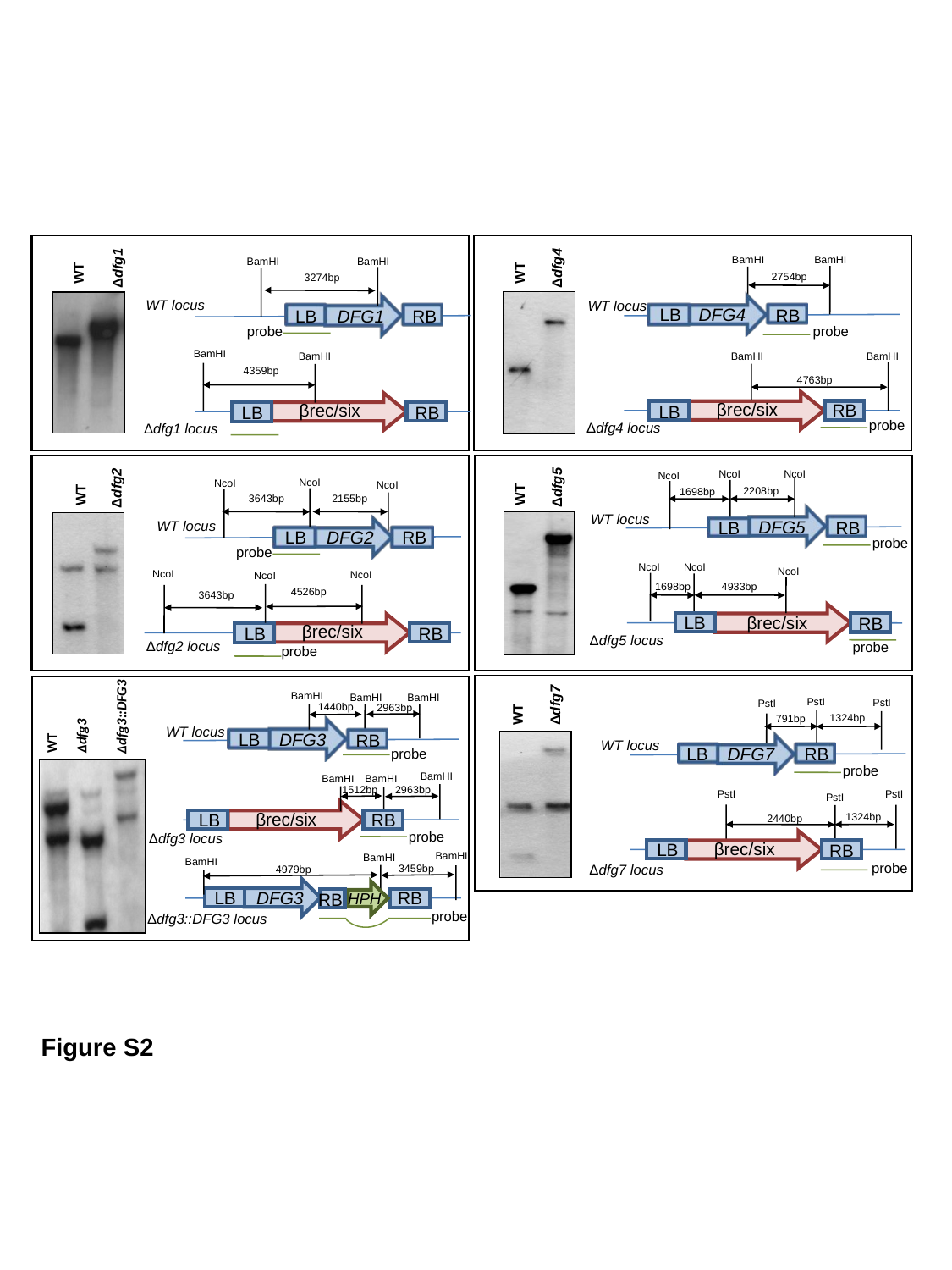

BamHI
BamHI
Δdfg4
WT
2754bp
WT locus
LB
DFG4
RB
probe
BamHI
BamHI
4763bp
RB
LB
βrec/six
probe
Δdfg4 locus
BamHI
BamHI
RB
DFG1
LB
probe
BamHI
βrec/six
LB
RB
BamHI
Δdfg1
WT
3274bp
WT locus
4359bp
Δdfg1 locus
NcoI
NcoI
NcoI
Δdfg5
2208bp
1698bp
WT
WT locus
DFG5
LB
RB
probe
NcoI
NcoI
NcoI
1698bp
4933bp
βrec/six
LB
RB
Δdfg5 locus
probe
NcoI
NcoI
NcoI
Δdfg2
WT
3643bp
2155bp
WT locus
RB
DFG2
LB
probe
NcoI
NcoI
NcoI
4526bp
3643bp
βrec/six
RB
LB
Δdfg2 locus
probe
BamHI
BamHI
BamHI
1440bp
2963bp
WT locus
DFG3
LB
RB
probe
Δdfg3::DFG3
Δdfg3
WT
BamHI
BamHI
BamHI
1512bp
2963bp
βrec/six
LB
RB
probe
Δdfg3 locus
PstI
PstI
Δdfg7
PstI
WT
1324bp
791bp
WT locus
DFG7
LB
RB
probe
PstI
PstI
PstI
1324bp
2440bp
βrec/six
LB
RB
probe
Δdfg7 locus
BamHI
BamHI
BamHI
3459bp
4979bp
DFG3
RB
LB
RB
HPH
probe
Δdfg3::DFG3 locus
Figure S2
