# Supplementary material for: The Glycosylphosphatidylinositol-Anchored DFG Family Is Essential for the Insertion of Galactomannan into the β-(1,3)-Glucan–Chitin Core of the Cell Wall of Aspergillus fumigatus
Source: mSphere. 2019 Jul 31;4(4):e00397-19. doi: 10.1128/mSphere.00397-19 (PMC6669337; doi:10.1128/mSphere.00397-19)
Supplement: TABLE S2 [file mSphere.00397-19-st002.docx]

**Table S2:** *Aspergillus fumigatus* **s**trains used in this study

| **Strain** | **Genotype** | **Source** |
| --- | --- | --- |
| WT | CEA17 Δ akuB^KU80^ | Da silva Ferreira et al, 2006 |
| *Δdfg1* | CEA17_ΔakuB^KU80^Δ*DFG1:: six-β-rec-hygroR-six* | This study |
| *Δdfg2* | CEA17_ΔakuB^KU80^Δ*DFG2:: six-β-rec-hygroR-six* | This study |
| *Δdfg3* | CEA17_ΔakuB^KU80^Δ*DFG3:: six-β-rec-hygroR-six* | This study |
| *Δdfg4* | CEA17_ΔakuB^KU80^Δ*DFG4:: six-β-rec-hygroR-six* | This study |
| *Δdfg5* | CEA17_ΔakuB^KU80^Δ*DFG5:: six-β-rec-hygroR-six* | This study |
| *Δdfg7* | CEA17_ΔakuB^KU80^Δ*DFG7:: six-β-rec-hygroR-six* | This study |
| *Δdfg5/2* | CEA17_ΔakuB^KU80^Δ*DFG5:: six/*Δ*DFG2::six-β-rec-hygroR-six* | This study |
| *Δdfg5/2/1* | CEA17_ΔakuB^KU80^Δ*DFG5::six/*Δ*DFG2::six/*Δ*DFG1::six-β-rec-hygroR-six* | This study |
| *Δdfg5/2/1/3* | CEA17_ΔakuB^KU80^Δ*DFG5::six/*Δ*DFG2::six/*Δ*DFG1::six/*Δ*DFG3::six-β-rec-hygroR-six* | This study |
| *Δdfg5/2/1/4* | CEA17_ΔakuB^KU80^Δ*DFG5::six/*Δ*DFG2::six/*Δ*DFG1::six/*Δ*DFG4::six-β-rec-hygroR-six* | This study |
| *Δdfg5/2/1/3/4* | CEA17_ΔakuB^KU80^Δ*DFG5::six/*Δ*DFG2::six/*Δ*DFG1::six/*Δ*DFG4::/*Δ*DFG3::six-β-rec-hygroR-six* | This study |
| *Δdfg5/2/1/3/4/7* | CEA17_ΔakuB^KU80^Δ*DFG5::six/*Δ*DFG2::six/*Δ*DFG1::six/*Δ*DFG3::six/*Δ*DFG4::six/six-β-rec-hygroR-six/*Δ*DFG7::PHLE* | This study |
| *Δdfg3::DFG3* | CEA17_ΔakuB^KU80^Δ*DFG3::DFG3*::*six* | This study |
