# Supplementary figures and images for: The Glycosylphosphatidylinositol-Anchored DFG Family Is Essential for the Insertion of Galactomannan into the β-(1,3)-Glucan–Chitin Core of the Cell Wall of Aspergillus fumigatus
Source: mSphere. 2019 Jul 31;4(4):e00397-19. doi: 10.1128/mSphere.00397-19 (PMC6669337; doi:10.1128/mSphere.00397-19)

## Slide 1
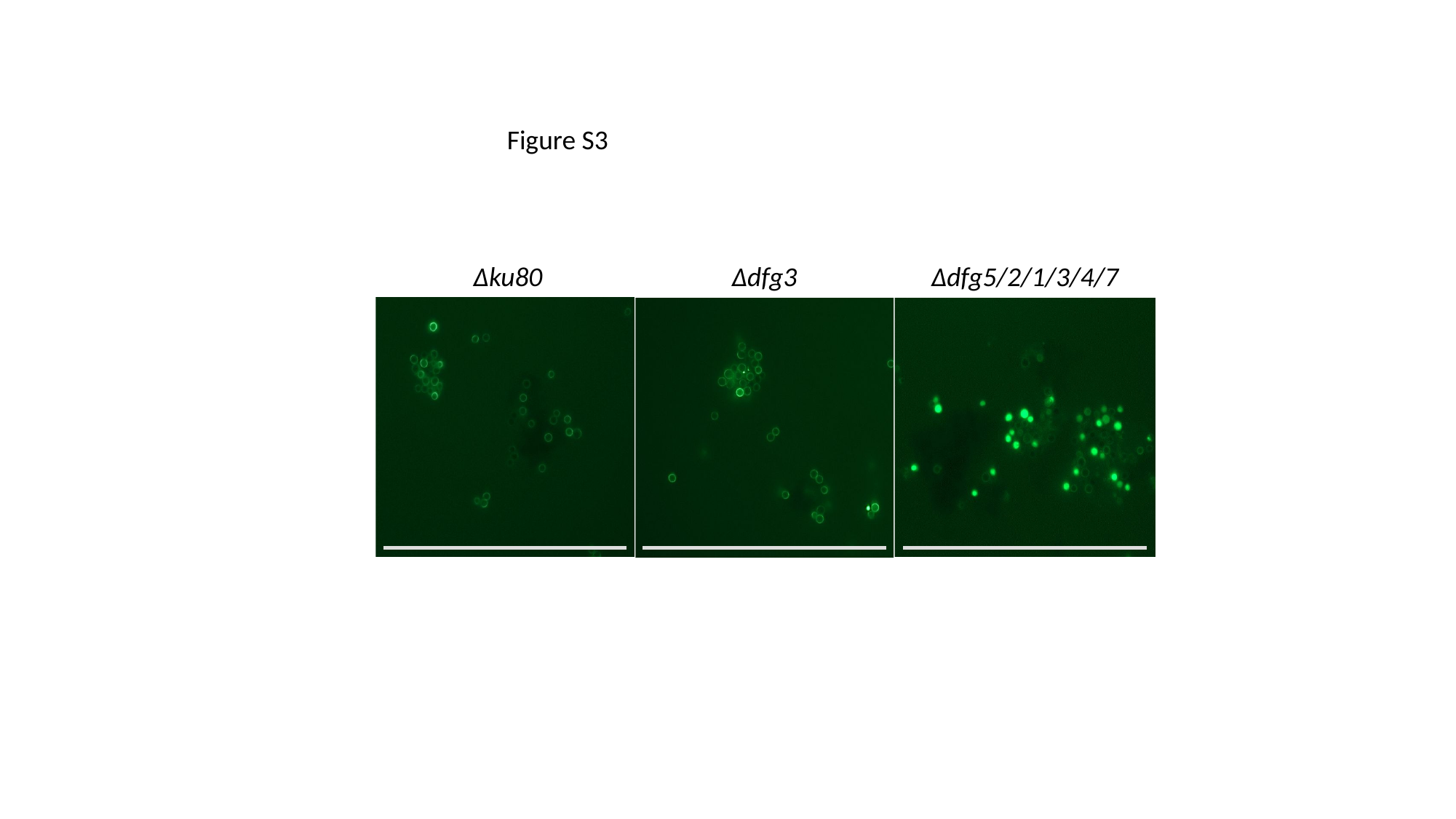

Figure S3
∆dfg5/2/1/3/4/7
∆ku80
∆dfg3

Supplement: FIG S3 [file mSphere.00397-19-sf003.pptx]

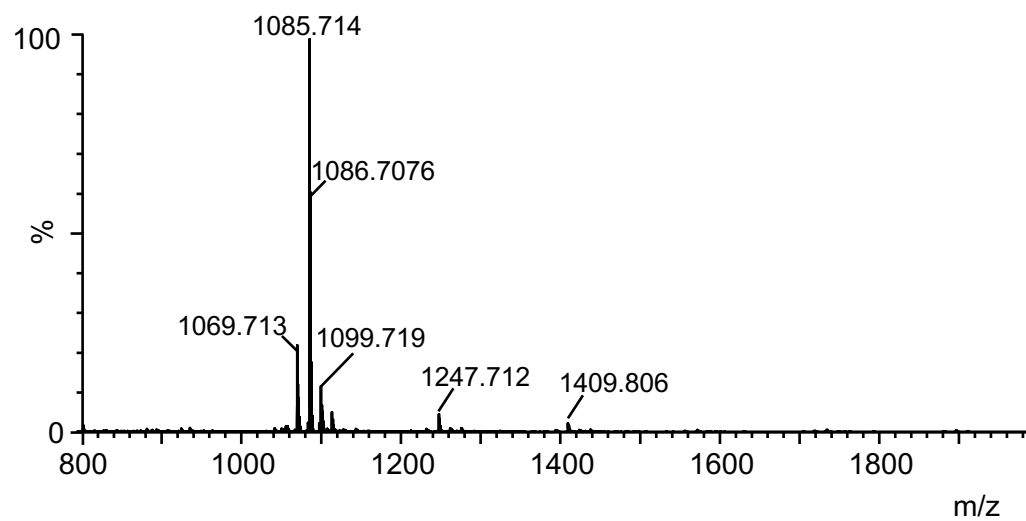

**Figure S4**

Supplement: FIG S4 [file mSphere.00397-19-sf004.pdf]

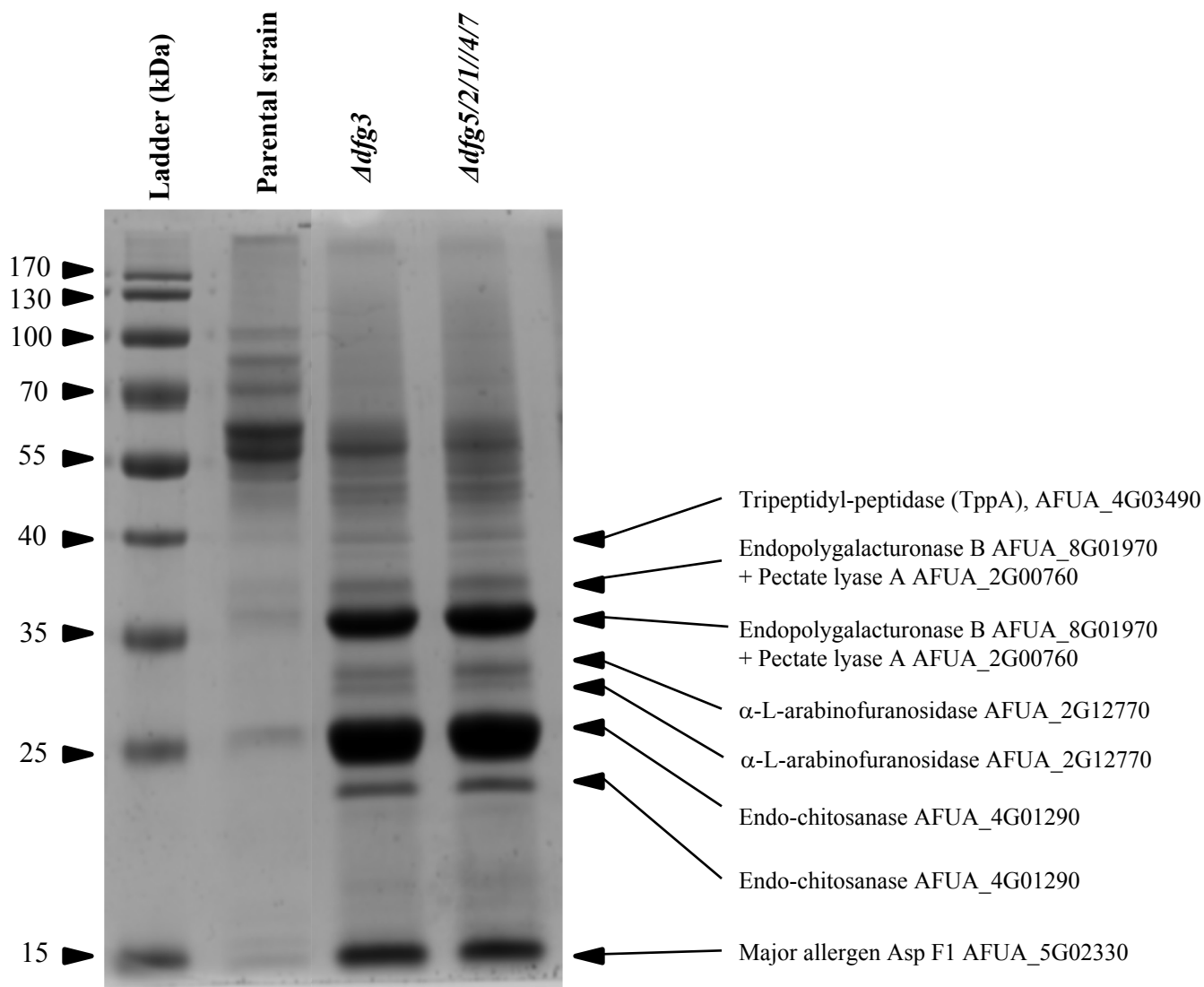

**Figure S5**

Supplement: FIG S5 [file mSphere.00397-19-sf005.pdf]
