# Supplementary material for: The Glycosylphosphatidylinositol-Anchored DFG Family Is Essential for the Insertion of Galactomannan into the β-(1,3)-Glucan–Chitin Core of the Cell Wall of Aspergillus fumigatus
Source: mSphere. 2019 Jul 31;4(4):e00397-19. doi: 10.1128/mSphere.00397-19 (PMC6669337; doi:10.1128/mSphere.00397-19)
Supplement: TEXT S1 [file mSphere.00397-19-s0001.docx]

**Supplementary methods. Production of recombinant ScDcw1p and enzymatic assays**

**Production of recombinant ScDcw1p**

*Sc*Dcw1p was produced as an MBP-fusion protein using the vector pMAL-cRI in *E. coli* BL21-Gold cells for 40 h at 20 °C in 5 L baffled flasks with 2 L LB-medium (10 g/L Tryptone, 10 g/L NaCl, 5 g/L yeast extract, 400 µL 10 M NaOH per liter medium) supplemented with 12.5 g/L lactose for autoinduction (1). The cells were harvested (20 min, 4 °C, 3000 × *g*) and the pellet of 2 L culture was resuspended in 30 mL lysis buffer (300 mM NaCl, 20 mM HEPES, pH 7.5). After cell lysis using a french press and centrifugation (20 min, 4 °C, 40000 × *g*), the cell free lysate was loaded on 15 mL Amylose column equilibrated with 10 CV of lysis buffer. Subsequently, the column was washed with 100 mL lysis buffer and finally the protein was eluted with 30 mL elution buffer (lysis buffer with 10 mM maltose). The solution was concentrated to 1 mL with a spin concentrator (MWCO 30 kDa) and loaded on a HiLoad 26/600 Superdex S200 column (GE Healthcare), which was equilibrated with lysis buffer. The protein eluted as a single peak. Its respective fractions were collected and checked on 12% SDS-PAGE.

Sequence of the MBP-*Sc*Dcw1fusion protein

MKTEEGKLVIWINGDKGYNGLAEVGKKFEKDTGIKVTVEHPDKLEEKFPQVAATGDGPDIIFWAHDRFGGYAQSGLLAEITPDKAFQDKLYPFTWDAVRYNGKLIAYPIAVEALSLIYNKDLLPNPPKTWEEIPALDKELKAKGKSALMFNLQEPYFTWPLIAADGGYAFKYENGKYDIKDVGVDNAGAKAGLTFLVDLIKNKHMNADTDYSIAEAAFNKGETAMTINGPWAWSNIDTSKVNYGVTVLPTFKGQPSKPFVGVLSAGINAASPNKELAKEFLENYLLTDEGLEAVNKDKPLGAVALKSYEEELAKDPRIAATMENAQKGEIMPNIPQMSAFWYAVRTAVINAASGRQTVDEALKDAQTNSSSVPSSLVIEGRISEFVELDLDNYESLQNATSLIAYGLMDYYTGNQYGKTVGMFSDPYYWWEAGGAWGCMLDYWFFMDNDTYNDEIIAAMIHQAGDDNDYIPLNQSTTEGNDDQAFWGIAAMTAAERNFTNPPENEPQWLYLAQAVFNTMALRWDADSCGGGLRWQIFVWNSGYDYKNTVSNGALFHIAARLARYTGNQTYVDWAEKVYEWMVGVNLISNGTYKYVYDGVSIDDNCTKVTSYQWTYNQGLLLAGSAYLYNFTGSDLWHTRTKEFLNASQVFFHDGIVYEAACQGPNSCNTDQRSFKAYFARFLGVTAQLVPETRNQIMSWLNTSAIAAAKSCSGGTDGHTCGLNWFNGTWDGMYGLGEQMSALEVMVNTRALDKPAPYTAENGGSSVGDGAAGTQAQPTNLAPLNITKGSK

116

66

45

35

25

MW markers


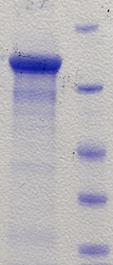


SDS-PAGE of purified MBP-*Sc*Dcw1p

**Mannosidase and transferase assays**

Mannosidase activities was tested using several substrates : pNP-α-mannoside (Sigma), α-1,6-mannobioside (Sigma), 4-methylumbelliferyl-α-(1,6)-mannobioside (produced as previously described (2), LGM (3) and mannan from ∆mn2 *S. cerevisiae* mutant. Briefly, 2 µM of purified rDcw1p were incubated at pH 5 with 1 mg/ml of substrate in a final volume of 100 µl at 30°C for 20h. Mannosidase activity was estimated by measuring the produced sugar reducing end with the *p*-hydroxybenzoyic acid hydrazide reagent (4) or the release of *p*-nitrophenyl. The assay with 4-methylumbelliferyl-α-(1,6)-mannobioside has been performed as previously described for bacterial GH76 member (5).

Putative galactomannan transfer onto β-glucan activity was tested by using the lipogalactomannan as donor and a glucan acceptor substrate. Two couples of substrates were performed. In the first couple, the lipogalactomannan was labelled with the fluorescent coumarin group and the AI cell wall fraction from *S. cerevisiae*. In the second, the purified LGM and soluble 2-aminopyridine conjugated laminarioligosaccharides were used as donor and acceptor substrates respectively.

Labelling of the LGM : 3.5 mg of LGM were partially oxidized in 1.75 ml of 100 mM NaOAc pH 5.5 by 43.5 µl of 40mM *m*-IO_4_Na on water-ice for 30 minutes. Oxidation was stopped by addition of 40 µl of glycerol, then the mixture was dialyzed against water and freeze dried. 7-amino-4-methyl-coumarin (AMC) was then conjugated onto produced aldehyde functions of oxidized LGM by aminoreduction (6).

Laminarioligosaccharides were produced by acetolysis of curdlan as previously described. Labelling with 2-aminopydine (2-AP) was performed as described previously (7). Then Gel filtration chromatography on G25-Sephadex allowed to isolated fraction of 2-AP labelled laminariologosaccharide with a DP 10 up to 20.

Transfer test1 : 15µl of AI cell wall fraction (5 mg/ml) and 15 µl of AMC labelled LGM were incubated overnight at 37°C in 100 mM NaOAc pH 5 buffer (60 µl final volume) with 8 µM rDcw1p. Insoluble material was recovered by centrifugation (5 minutes, 10000 g) and extensively washed with 2% sodium carbonate. AMC conjugated LGM retained in the insoluble pellet was quantified with a spectrofluorimeter (λex 360 nm ; λem 460 nm ; Tecan).

Transfer test 2: 10 µl of LGM (5 mg/ml) and 25 µl of 2AP-glucan (1 mg/ml) were incubated overnight a 30°C in 100 mM NaOAc pH 5 with 8 µM rDcw1p. The transfer product was analyzed by gel filtration chromatography on the Superdex 75 column (10/30 GE Healthcare) as described above. 2-AP containing molecules were detected by fluorescence (λex 320 nm ; λem 400 nm ; detector Merck Lachrom).

**
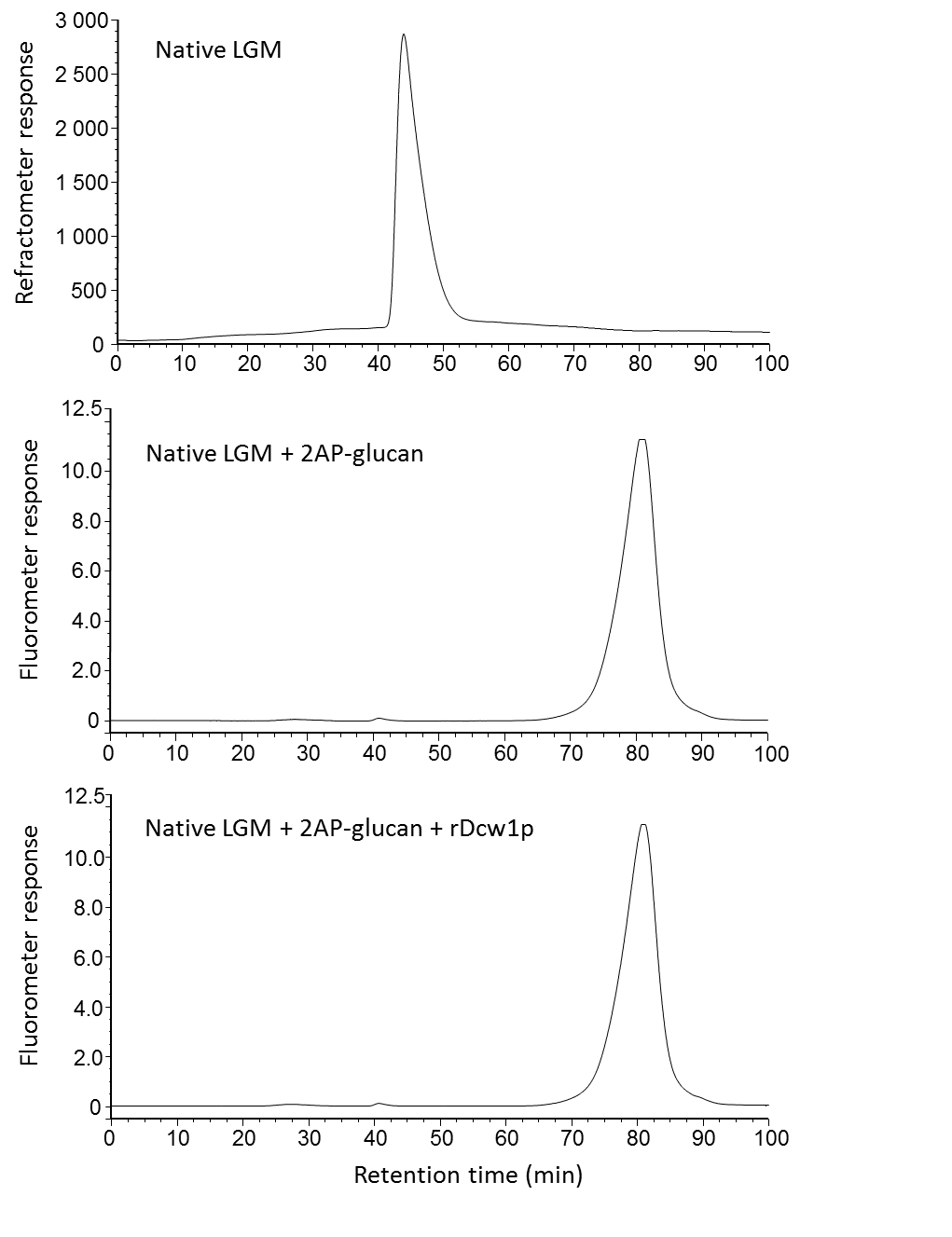
**

**Gel filtration chromatography of the transfer product assay**. Gel filtration was performed using a Superdex 75 column (GE Healthcare, 10/30) at eluted with 0.15 M ammonium acetate pH 4 at 0.2 ml/min. LGM was used as donor substrate; control elution was performed with a refractometer detector. The acceptor substrate is 2-aminopyridine conjugated laminarioligosaccharides that are detected by fluorescence. The absence of fluorescence peak coeluted with the LGM in presence of rDcw1p showed the absence of transfer.

**References**

1. [Studier FW](https://www.ncbi.nlm.nih.gov/pubmed/?term=Studier%20FW%5BAuthor%5D&cauthor=true&cauthor_uid=15915565).2005. Protein production by auto-induction in high density shaking cultures. [Protein Expr Purif.](https://www.ncbi.nlm.nih.gov/pubmed/?term=Studier%2C+Protein+Expr.+Purif.+(2005)+41(1)%3A207%E2%80%93234%2C.) 41:207-34.
2. Henry C, Fontaine T, Heddergott C, Robinet P, Aimanianda V, Beau R, Beauvais A, Mouyna I, Prevost MC, Fekkar A, Zhao Y, Perlin D, Latgé JP. 2016. [Biosynthesis of cell wall mannan in the conidium and the mycelium of Aspergillus fumigatus.](https://www.ncbi.nlm.nih.gov/pubmed/27603677) Cell Microbiol18:1881-1891

# [Costachel C](https://www.ncbi.nlm.nih.gov/pubmed/?term=Costachel%20C%5BAuthor%5D&cauthor=true&cauthor_uid=16204227), [Coddeville B](https://www.ncbi.nlm.nih.gov/pubmed/?term=Coddeville%20B%5BAuthor%5D&cauthor=true&cauthor_uid=16204227), [Latgé JP](https://www.ncbi.nlm.nih.gov/pubmed/?term=Latg%C3%A9%20JP%5BAuthor%5D&cauthor=true&cauthor_uid=16204227), [Fontaine T](https://www.ncbi.nlm.nih.gov/pubmed/?term=Fontaine%20T%5BAuthor%5D&cauthor=true&cauthor_uid=16204227). 2005 Glycosylphosphatidylinositol-anchored fungal polysaccharide in *Aspergillus fumigatus*. 280: 39835-39842

1. Fontaine T, Hartland RP, Diaquin M, Simenel C, Latgé JP. 1997 [Differential patterns of activity displayed by two exo-beta-1,3-glucanases associated with the Aspergillus fumigatus cell wall.](https://www.ncbi.nlm.nih.gov/pubmed/9150209) J Bacteriol 179:3154-63.
2. Striebeck A, Robinson DA, Schüttelkopf AW, van Aalten DMF. 2013. Yeast Mnn9 is both a priming glycosyltransferase and an allosteric activator of mannan biosynthesis. Open Biol 3:130022
3. [Bigge JC](https://www.ncbi.nlm.nih.gov/pubmed/?term=Bigge%20JC%5BAuthor%5D&cauthor=true&cauthor_uid=7503412), [Patel TP](https://www.ncbi.nlm.nih.gov/pubmed/?term=Patel%20TP%5BAuthor%5D&cauthor=true&cauthor_uid=7503412), [Bruce JA](https://www.ncbi.nlm.nih.gov/pubmed/?term=Bruce%20JA%5BAuthor%5D&cauthor=true&cauthor_uid=7503412), [Goulding PN](https://www.ncbi.nlm.nih.gov/pubmed/?term=Goulding%20PN%5BAuthor%5D&cauthor=true&cauthor_uid=7503412), [Charles SM](https://www.ncbi.nlm.nih.gov/pubmed/?term=Charles%20SM%5BAuthor%5D&cauthor=true&cauthor_uid=7503412), [Parekh RB](https://www.ncbi.nlm.nih.gov/pubmed/?term=Parekh%20RB%5BAuthor%5D&cauthor=true&cauthor_uid=7503412). 1995 Nonselective and efficient fluorescent labeling of glycans using 2-amino benzamide and anthranilic acid. [Anal Biochem.](https://www.ncbi.nlm.nih.gov/pubmed/?term=Bigge+et+al.%2C+Anal.+Biochem.+(1995)+230%3A229-238) 230:229-38.
4. [Kuraya N](https://www.ncbi.nlm.nih.gov/pubmed/?term=Kuraya%20N%5BAuthor%5D&cauthor=true&cauthor_uid=1429500), [Hase S](https://www.ncbi.nlm.nih.gov/pubmed/?term=Hase%20S%5BAuthor%5D&cauthor=true&cauthor_uid=1429500). 1992 Release of O-linked sugar chains from glycoproteins with anhydrous hydrazine and pyridylamination of the sugar chains with improved reaction conditions. [J Biochem.](https://www.ncbi.nlm.nih.gov/pubmed/?term=Karuya+and+Hase+J+Biochem.+(1992)+Jul%3B112(1)%3A122-6.)112:122-6.
