# Supplementary material for: The Glycosylphosphatidylinositol-Anchored DFG Family Is Essential for the Insertion of Galactomannan into the β-(1,3)-Glucan–Chitin Core of the Cell Wall of Aspergillus fumigatus
Source: mSphere. 2019 Jul 31;4(4):e00397-19. doi: 10.1128/mSphere.00397-19 (PMC6669337; doi:10.1128/mSphere.00397-19)
Supplement: TABLE S3 [file mSphere.00397-19-st003.docx]

**Table S3 :** Primer list used in this study

| **NAME** | **SEQUENCE 5’🡪 3’** | **USE** |
| --- | --- | --- |
| For FG *DFG*1 | GCCAAGCTTGCATGCCGATATCGGTGGCCCAATCCGAACGT | Construction of the deletion cassette *DFG*1 |
| Rev FG *DFG*1 | CCTGAGTGATGCTTTGCGACGTCACTGCGG |  |
| For FD *DFG*1 | TGGTCCATCTAGTGCTGAGGCGTCCGGAGGAGC |  |
| Rev FD *DFG*1 | AATTCGAGCTCGGTACGATATCGGACGGACAGTGCGGAGG |  |
| For FG *DFG*2 | GCCAAGCTTGCATGCCTGCGCACGATTCGTTCCGCTCGGTC | Construction of the deletion cassette *DFG*2 |
| Rev FG *DFG*2 | GGACCTGAGTGATGCGGTCATGCAGGTCCGACG |  |
| For FD *DFG*2 | TGGTCCATCTAGTGCGCCTGATTGGAGGGCCTG |  |
| Rev FD *DFG*2 | AATTCGAGCTCGGTACTGCGCACGAGCAGCAAAGCCCAAG |  |
| For FG *DFG*3 | GCCAAGCTTGCATGCCTGCGCACCAGTCGCACCGACGATG | Construction of the deletion cassette *DFG*3 |
| Rev FG *DFG*3 | GGACCTGAGTGATGCGGGGCAAGGCAGTGATGC |  |
| For FD *DFG*3 | TGGTCCATCTAGTGCCGGCGTGATTTGGTGAGG |  |
| Rev FD *DFG*3 | AATTCGAGCTCGGTACTGCGCATGGTGACAGGGCCAGAGC |  |
| For FG *DFG*4 | GCCAAGCTTGCATGCCTGCGCACTGTAAGACGCGAGGCCG | Construction of the deletion cassette *DFG*4 |
| Rev FG *DFG*4 | GGACCTGAGTGATGCCGGACAATGCGACACCCA |  |
| For FD *DFG*4 | TGGTCCATCTAGTGCCTCGGCATTCGACCACCA |  |
| Rev FD *DFG*4 | AATTCGAGCTCGGTACTGCGCATCCGCAATCATGGATGGG |  |
| For FG *DFG*5 | GCCAAGCTTGCATGCCTGCGCATGACGCAAAGACAGGGAAGC | Construction of the deletion cassette *DFG*5 |
| Rev FG *DFG*5 | GGACCTGAGTGATGCCACCGTCGCCTGGATATTCCC |  |
| For FD *DFG*5 | TGGTCCATCTAGTGCAGACAATGCGGAACCATCCG |  |
| Rev FD *DFG*5 | AATTCGAGCTCGGTACTGCGCAGCCAACAGCAACAGCCTCAT |  |
| For FG *DFG*7 | CCGTCTCTGCTGTTCTCTGCT | Construction of the deletion cassette *DFG*7 |
| Rev FG *DFG*7 | TTCTCCTTCGCTTACTGCCGCCACAACGGTACAGACTTCG |  |
| For FD *DFG*7 | ACAACGTCGTGACTGGGAAACCTGCCAGAGGCTCCTAATG |  |
| Rev FD *DFG*7 | GGTACTGGTCCCACACCTTG |  |
| For PCR3 | CTTTGGGAACGGGGCGATAT |  |
| RevPCR3 | CAGGTTGCCACCACTGTTTG |  |
| ForPhleo | CGGCAGTAAGCGAAGGAGAA |  |
| RevPhleo | TTTCCCAGTCACGACGTTGT |  |
| For ORF *DFG*3 | GCCAAGCTTGCATGCCATGCGCATGCTGAGTTTGCG | Construction of the complementation cassette *DFG*3 |
| RevORF*DFG*3 | TGTTCCTCGACTTCCCACCATATTGGGTAGATGCG |  |
| For FDC *DFG*3 | CCAGCTTGTGTTCCCCTCCCTGCGATTCCCATCTC |  |
| Rev FDC *DFG*3 | AATTCGAGCTCGGTACTGCGCACGTTCGCTGGGTTGATTATC |  |
| ForHygro | GGAAGTCGAGGAACAGACCG |  |
| RevHygro | GGGAACACAAGCTGGCAGTC |  |
| For*DFG*1 | GACACTGGAGGTACGAGCAAGAG | *DFG*1 primers for  q-PCR analysis |
| Rev*DFG*1 | GTGACTGGCTGCGGTTGAGG |  |
| For*DFG*2 | GCGATGCGAACAATCAGATGGG | *DFG*2 primers for  q-PCR analysis |
| Rev*DFG*2 | CCGTAGCCGGAACTATGAGAGGAA |  |
| For*DFG*3 | CGGCTTCACTCCTGGTCTGG | *DFG*3 primers for  q-PCR analysis |
| Rev*DFG*3 | GGTGTCAATCTGATGCTCCTTGC |  |
| For*DFG*4 | GATGGACGACGGGCGAGTTC | *DFG*4 primers for  q-PCR analysis |
| Rev*DFG*4 | TCCACCGCATGTTCCTCTTG |  |
| For*DFG*5 | GCGTGCGGGCTGAAATGG | *DFG*5 primers for  q-PCR analysis |
| Rev*DFG*5 | CGGTGGCATCGGTGACTGG |  |
| For*DFG*6 | ACGGGTGGACGGGTATGGAG | *DFG*6 primers for  q-PCR analysis |
| Rev*DFG*6 | AGTCGGGTTGCTGGTGCTATTC |  |
| For*DFG*7 | CGGACGGCAAGGAGCATCAG | *DFG*7 primers for  q-PCR analysis |
| Rev*DFG*7 | CCCAGCAGTAAGCACAAAGATCA |  |
| For EF1α | CCATGTGTGTCGAGTCCTTC | *TEF1*primers for  q-PCR analysis |
| Rev EF1 α | GAACGTACAGCAACAGTCTGG |  |
|  |  |  |
| 1975 DCW1 3’R | ACATGCATGCAGGAAACCATGTAAGCGATGAATAT | Construction of *DCW1* deletion plasmid |
| 1976 DCW1 3’F | GGGGTACCTGCAGAACTTATGAAAGCTTAACATTT |  |
| 1977 DCW1 5’R | GGGGTACCTTTTATGTGTTCGTTTTTAAAACAGAC |  |
| 1978 DCW1 5’F | CCCCAAGCTTAGATGAACTTGAACTTAAGATGATC |  |
| 2306 DCW1 5’ +1500 | TCGTTTAAATTCAATTGGAACTGTA | Construction of p413TEF.*DCW1* plasmid |
| 2307 DCW1 3’ -1500 | TTCAAACAAAATTCGTTCGATATTA |  |
| 2309 DCW1 5’BamH1 | ACTAGTGGATCCATGCTAGTAAATAAAGTGA | Construction of p413TEF.*dcw1^ts^* plasmid |
| 2310 DCW1 3’Xho1 | ACATGACTCGAGGTCGACGGTATCGATAA |  |
|  |  |  |
|  |  |  |
|  |  |  |
